# Unraveling the Molecular Links between Fine Particulate Matter Exposure and Early Birth Risks in African American Mothers: A Metabolomics Study in the Atlanta African American Maternal-Child Cohort

**DOI:** 10.1021/acs.est.5c02071

**Published:** 2025-05-29

**Authors:** Zhenjiang Li, Anne L. Dunlop, Jeremy A. Sarnat, Anke Hüls, Stephanie M. Eick, Audrey Gaskins, Howard Chang, Armistead Russell, Youran Tan, Haoran Cheng, Dana Boyd Barr, Alicia K. Smith, Carmen Marsit, Dean P. Jones, Donghai Liang

**Affiliations:** † Gangarosa Department of Environmental Health, Rollins School of Public Health, 1371 Emory University, Atlanta, Georgia 30322, United States; ‡ Department of Gynecology and Obstetrics, School of Medicine, Emory University, Atlanta, Georgia 30322, United States; § Department of Epidemiology, Rollins School of Public Health, Emory University, Atlanta, Georgia 30322, United States; ∥ Department of Biostatistics, Rollins School of Public Health, Emory University, Atlanta, Georgia 30322, United States; ⊥ School of Civil and Environmental Engineering, 1372 Georgia Institute of Technology, Atlanta, Georgia 30332, United States; # Department of Medicine, School of Medicine, Emory University, Atlanta, Georgia 30322, United States

**Keywords:** high-resolution metabolomics, high-dimensional mediation analysis, fine particulate matter, preterm birth, early term birth, energy metabolism, amino acid metabolism, minority health, environmental justice

## Abstract

In the United States, African Americans (AA) are disproportionately exposed to elevated levels of ambient fine particulate matter (PM_2.5_) while suffering from the highest rates of early births. To elucidate the largely unknown underlying mechanism, we analyzed serum metabolomics from 330 participants in the Atlanta AA Maternal-Child Cohort and performed high-throughput mediation analysis to identify intermediate metabolites and pathways linking PM_2.5_ to early births. Energy-metabolism-related metabolites (carnitine and adenosine triphosphate), along with lysoPE(20:3) and acetylcysteine, were both associated with PM_2.5_ exposure and elevated early birth risks. Perturbations in protein digestion and absorption and aromatic amino acid (phenylalanine, tyrosine, and tryptophan) metabolism may potentially mediate the associations between PM_2.5_ and early births. We identified significant indirect effects of cortexolone (Proportion mediated: −11.8%) and lysoPE(20:3) (9.4%) in mediating the relationship between PM_2.5_ and early births. Our findings might aid in early birth prevention among AA communities by providing novel insights into the underlying biological mechanism.

## Graphical abstract

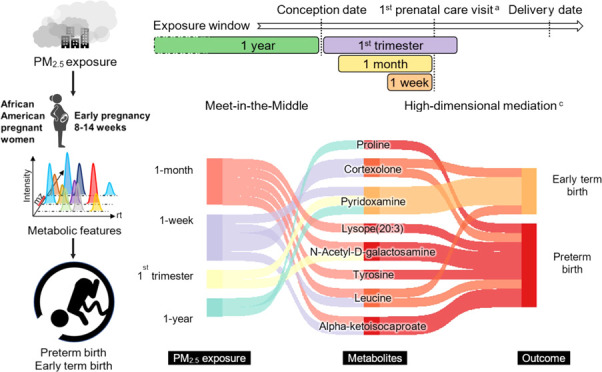

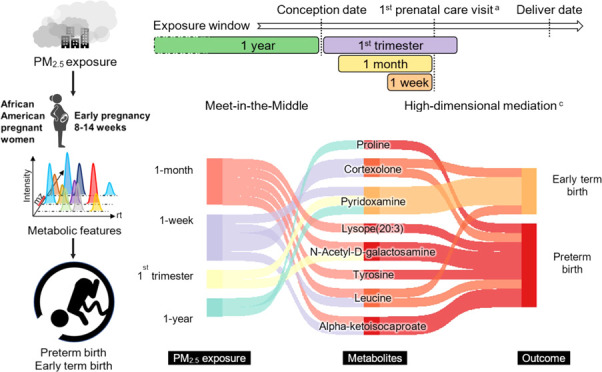

## Introduction

Ambient fine particulate matter (PM_2.5_) is a significant contributor to public health burden with adverse health effects well characterized across all life stages.^1–5^ Notably, pregnant individuals and fetuses are more vulnerable to PM_2.5_ exposure compared to the general population.^6^ Existing evidence shows that prenatal exposure to PM_2.5_ associates with a series of adverse birth outcomes, including preterm (PTB) and early term birth (ETB),^6^ defined as being born prior to 37 weeks and 37–39 weeks gestation, respectively.^7^ PTB is a major contributor to childhood morbidity and mortality, responsible for 17.7% of global deaths among children under five years of age,^8^ making it the leading cause of under-five mortality worldwide. PTB is also linked to complications such as respiratory distress syndrome, cerebral palsy, and long-term risks of noncommunicable diseases due to disruption of fetal organ development.^9^ ETB, although less severe, has also been associated with increased neonatal morbidity and developmental challenges.^10^ Both outcomes represent a continuum of shortened gestation and have been linked to environmental exposures including PM_2.5_. Globally, approximately 10% of PTB cases are estimated to be attributable to ambient PM_2.5_ exposure, with the highest burden in sub-Saharan Africa.^11^

Communities of color and low-income communities in the United States (U.S.), especially African Americans (AA), experience disproportionately higher rates of PTB and ETB, highlighting that health disparities begin *in utero*.^12,13^ Additionally, communities of color in metropolitan areas across the U.S. are exposed to significantly worse long- and short-term PM_2.5_ pollution.^14^ Although a significant body of research has explored the association between prenatal PM_2.5_ exposure and PTB, reported findings have been limited among AA communities despite their disproportionate exposures.^15^ Further, some uncertainty in the existing data is due to the use of different exposure windows (before and during pregnancy) and the complex biological mechanisms involved in the etiology of PTB and ETB.

High-resolution metabolomics has emerged as a powerful analytic platform in environmental health research, with demonstrated utility for characterizing the biological perturbations in the human metabolome resulting from short- and long-term exposures to air pollution.^16^ High-resolution metabolomics enables the identification and quantification of thousands of metabolic features, offering a comprehensive view of both exogenous exposures and endogenous processes in biospecimens.^17^ Unlike targeted methods, which focus on measuring a limited predefined set of metabolites, high-resolution metabolomics provides an untargeted approach that allows for the discovery of novel biomarkers and the exploration of previously unrecognized biological pathways involved in disease etiology.^18^ This method is particularly valuable in environmental health research, where exposure mixtures, such as air pollution, can induce multifaceted biological responses.^18^ Previous studies, including our own, have successfully applied high-resolution metabolomics to uncover metabolic signatures associated with various environmental exposures,^19–25^ significantly advancing our understanding of the biological mechanisms underlying exposure-induced diseases. Understanding the mechanisms underlying the toxicity of PM_2.5_ exposure on risk of PTB and ETB is important for guiding policies and interventions to reduce risks in vulnerable populations.^17^ A previous study found that alterations of oxidative stress and inflammation-related pathways within the midpregnancy serum metabolome were associated with air pollution exposure among 160 U.S. mothers of multiple races.^26^ In addition, researchers have employed metabolomics to identify biomarkers and pathways predictive of PTB, many of which were also associated with PM_2.5_ exposures.^27–29^ These initial findings suggest that prenatal PM_2.5_ exposure may lead to an increased risk of PTB and ETB by altering levels of intermediate metabolites, such as pro-inflammatory factors.^6^ Mediation analysis is crucial for uncovering how endogenous metabolites mediate the relationship between PM_2.5_ exposure and early births. Specifically, identifying these metabolomic mediators in observational studies can strengthen the causal link and provide comprehensive insights into the biological mechanisms.^30^ However, only two epidemiological studies have explored metabolomics as intermediate variables or mediators of environmental pollution’s impact on reproductive or birth outcomes,^31,32^ and none have focused on the effects of prenatal PM_2.5_ exposure on early births.

To address these critical knowledge gaps, we conducted a comprehensive metabolome-wide association and high-throughput mediation study in the Atlanta AA Maternal-Child Cohort.^33^ We used advanced high-resolution metabolomics and mediation analyses to identify metabolic perturbations (i.e., altered metabolites and biological pathways) that mediate the association of exposures to ambient PM_2.5_ at four critical exposure time windows with the risks of PTB and ETB.

## Methods

### Study Population

The current analysis included study participants enrolled in the Atlanta AA Maternal-Child Cohort.^33,34^ Briefly, since 2014, this prospective cohort has recruited pregnant individuals presenting for prenatal care at clinics of Emory Midtown Hospital (private) and Grady Memorial Hospital (publicly funded) who met the following criteria: self-reported as U.S.-born and of African American or Black race with age between 18 and 40 years, without chronic medical conditions, with a singleton pregnancy estimated to be between 6 and 17 weeks of gestation (verified by medical record). No other exclusion criteria were applied regarding pregnancy complications. Health data were collected via questionnaires and medical record abstraction. Blood samples were obtained via venipuncture at the enrollment visit (targeting 6–17 weeks) and processed to obtain serum; aliquots of serum were stored at −80 °C until metabolomic assays were performed. Additional details regarding recruitment and enrollment are provided elsewhere.^33^ In total, we analyzed data from 330 participants with metabolomics data available at the enrollment visit, enrolled between March 2014 and May 2018. This study was approved by the Emory University Internal Review Board, and written informed consent was obtained from all study participants.

### Gestational Age at Birth Outcomes

Gestational age at birth in completed gestational weeks was abstracted from medical records and was based upon the best obstetrical estimate, following the American College of Obstetrics and Gynecology (ACOG) guidelines,^31,35^ considering the date of delivery in relation to the estimated date of confinement established by the first prenatal visit.^36^ Considering completed gestational weeks, births were classified as PTB (>20 and <37 weeks), ETB (≥37 and <39 weeks), and full-term birth (FTB, ≥39 weeks).^7^ PTB and ETB were the primary early birth outcomes of interest with FTB serving as the referent category.

### Air Pollution Exposure Assessment

Details of the ambient air pollution exposure assignment and the specific model used have been previously published.^37^ Briefly, the spatiotemporally resolved air quality model was processed in two stages. First, a calibrated Research LINE-source dispersion model for near-surface releases was used to estimate the annual-averaged traffic-related PM_2.5_ with a high spatial resolution. Second, a fusion modeling approach was used to integrate the traffic-related PM_2.5_ data and the publicly available Community Multiscale Air Quality (CMAQ) model, which is a chemical transport model simulating daily air pollution concentrations with a relatively low spatial resolution. Those estimates are calibrated by a Bayesian space-time downscaler model. As a result, the air quality model incorporated comprehensive chemistry and emission sources and created daily ambient PM_2.5_ data covering Metropolitan Atlanta from 2002 to 2018 with a spatial resolution of around 250 × 250 m. Then, we used daily estimated ambient PM_2.5_ concentrations at the participant’s geocoded residential address (which was collected at the first prenatal visit) as surrogates for individual exposure. Because exposure must precede the mediator (i.e., metabolic features), we selected four PM_2.5_ averaging periods: one-year prior to conception, the first trimester, and the one-month and one-week periods prior to the early pregnancy (6–17 week) collection of blood for serum metabolite measurement. Previous studies have shown that both long-term and short-term air pollution may influence the risk of preterm birth through distinct biological mechanisms.^38–40^ We considered the one-year average as a surrogate for long-term exposure, aligning with the air quality guideline of the World Health Organization, which defines long-term PM_2.5_ exposure based on annual averages.^41^ The first trimester window was selected based on previous findings identifying this period as a critical window of vulnerability for preterm birth.^42^ The short-term windows (one month and one week prior to blood draw) were included to align exposure timing with metabolite measurements, as metabolic responses to PM_2.5_ may vary depending on temporal proximity to the sampling date.^43,44^

### High-Resolution Metabolomics

Serum samples with unknown fasting status were analyzed using high-resolution liquid chromatography coupled with mass spectrometry (HR-LCMS, Thermo Scientific Q-Exactive HF) via an established protocol.^33,45^ Briefly, plasma samples were randomized into batches. In each batch, up to 12 quality control (QC) samples were added, including pooled human plasma samples and the National Institute of Standards and Technology (NIST) 1950 standard reference samples. All samples (i.e., study samples and QC samples) were run in triplicate and analyzed through two analytical columns, hydrophilic interaction liquid chromatography (HILIC) column with positive electrospray ionization (ESI), and C18 hydrophobic reversed-phase chromatography column with negative ESI. The metabolic features with mass-to-charge ratio (*m/z*), retention time (rt), and relative intensity were extracted by R packages *apLCMS* with *xMSanalyzer*,^46,47^ batch-corrected, averaged, and then transformed with the natural log for downstream analysis. Prior to downstream analysis, we evaluated feature quality using two metrics: the relative standard deviation (RSD) and missingness. We calculated the RSD as the standard deviation divided by the mean intensity of each feature across pooled human plasma samples from all batches. We assessed missingness in two ways: (1) the proportion of missing values among the study samples and (2) the proportion of missing values among pooled human plasma samples. To remove low-quality features while maximizing metabolome coverage, we excluded features that met either of the following conditions: (1) RSD > 50 and >10% missingness among pooled human plasma samples; (2) >90% missingness among study samples. As a result, 11,269 out of 13,616 and 9565 out of 11,900 metabolic features remained in the current analysis for the HILIC and C18 columns, respectively. The missing values were imputed by quantile regression imputation of left-censored data (QRILC) or random forest (RF).^48^ We classified the missing pattern [i.e., missing not at random (MNAR) vs missing at random (MAR)] using a second, correlated (Pearson’s correlation >0.5) auxiliary feature.^49^ Due to its correlation, we concluded that insights into the pattern of missing values of a given feature can be gained from the corresponding nonmissing observations of its auxiliary feature. The missing values of MNAR features were imputed by QRILC, while those of MAR features by RF, which was recommended in a previous study systematically comparing the imputation performance of different algorithms.^48^ Briefly, imputation methods for MS-based metabolomics data vary significantly by the type of missing values as this affects both imputation accuracy and statistical analysis. In a systematic comparison, Wei et al. found that RF imputation performed best for MAR data, while QRILC performed best for MNAR data.^48^ Metabolic features were matched to an in-house annotation database, resulting in 224 and 234 annotated metabolic features (resulting in 398 unique confirmed metabolites) for the HILIC and C18 columns, respectively. Specifically, metabolites were identified with confidence level 1 by comparison of *m*/*z*, retention time, and ion dissociation patterns to authentic chemical reference standards analyzed by using the identical method and instrument parameters via tandem mass spectrometry (MS/MS). The confidence system follows a five-level system of reporting standard proposed by Schymanski et al.^50^ For example, confidence level 1 indicated that the chemical identity of the metabolite is confirmed through comparison with an authentic standard. For metabolites without available reference standards, putative annotation was conducted by matching the accurate mass data to publicly available spectral libraries.

### Covariate Assessment

We determined the confounding structure based on literature review and our previous studies, which was illustrated via directed acyclic graphs (DAGs) (Figure S1). Individual-level demographic characteristics [maternal age and educational attainment (categorized as less than high school, high school, and some college or more)] were obtained via a standardized interview questionnaire. Infant sex (binary), parity (categorized as nulliparity, primiparity, and multiparity), and tobacco and marijuana use in the month prior to pregnancy (binary) were abstracted from the medical record. Maternal body mass index (BMI, kg/m^2^) was calculated using weight and height measured at the first visit. The meteorological covariates included the conception season (for long-term exposure) and averaged apparent temperature (for short-term exposure with the same time windows as air pollution estimates). The daily apparent temperature at the metro Atlanta airport was obtained from Automated Surface Observing System via R package *riem*.^51^ The gestational age at blood draw (i.e., at enrollment) in weeks was estimated by comparing the date of sampling to the estimated date of confinement based on the best obstetrical estimate (based on criteria of the American College of Obstetricians and Gynecologists).^35^ As shown in Figure S1A, we controlled for maternal age, maternal educational attainment, tobacco and marijuana use, and meteorological factors, as these covariates situated between PM_2.5_ exposure and metabolic features as biasing paths. We also adjusted for infant sex, maternal BMI, and gestational age at blood draw due to their known impact on maternal metabolism during pregnancy for the association between PM_2.5_ exposure and metabolic features.^52,53^ Similarly, we controlled for maternal age, maternal educational attainment, infant sex, maternal BMI, tobacco and marijuana use, parity, and gestational age at blood draw for the association between metabolic features and early birth. Maternal anatomy and physiology undergo substantial changes across gestational,^54^ and our participants had a relatively wide range of blood sample collection timing (6–17 weeks). To account for the variability in metabolic profiles associated with gestational age, we included gestational age at the blood draw as a covariate in all models. We considered the marital status as a proxy for potential spousal influence. However, comparisons of PM_2.5_ exposure levels and early birth outcomes (PTB and ETB) by marital status revealed no significant differences (Table S1), and thus we did not include it as a covariate in the final models. To assess potential confounding by pregnancy-related complications, we conducted sensitivity analyses adjusting for hypertensive disorders of pregnancy and gestational diabetes. Details on the collection of these pregnancy complications are available elsewhere.^55^ Briefly, the diagnosis of gestational hypertension or preeclampsia and gestational diabetes was determined based on medical record abstraction by trained clinical research staff, following the American College of Obstetrics and Gynecology (ACOG) guidelines.

### Statistical Analysis

We summarized maternal and newborn characteristics for participants stratified by gestational age in birth categories of interest. We tabulated the arithmetic means and standard deviations (SDs) of exposures and apparent temperature averages, and a descriptive statement of exposures was included in the main text. To identify the potential metabolic features mediating the association of PM_2.5_ with PTB and ETB, we adopted a parallel high-throughput mediation strategy using the Meet-in-the-Middle (MITM) and high-dimensional mediation analysis (HDMA) approaches (Figure 1).

**Figure 1:**
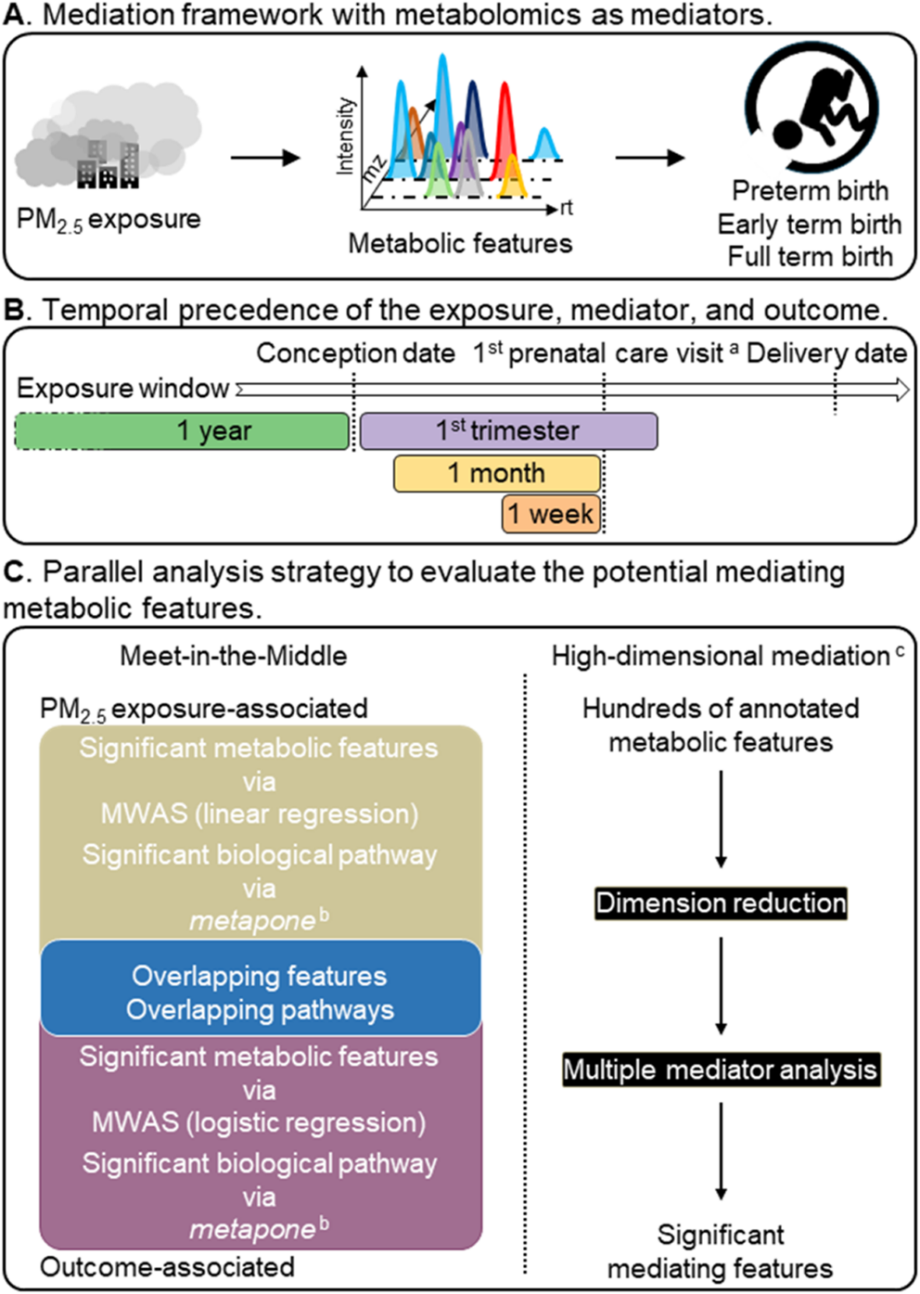
Graphical overview of the present study. (A) Mediation framework with metabolomics as mediators; (B) temporal precedence of the exposure, mediator, and outcome; (C) parallel analysis strategy to evaluate the potential mediating metabolic features. ^a^The date of first prenatal care visit varied by participating mothers from 6 to 17 gestational weeks. ^b^ *Metapone* was an R package to conduct pathway enrichment analysis for untargeted metabolomics data. ^c^Conducted by the R package *HIMA*. Mz, mass-to-charge ratio; rt, retention time; MWAS, metabolome-wide association study. Figure was created by the authors using Microsoft PowerPoint.

First, we conducted a metabolome-wide association study (MWAS) for exposures and outcomes separately and followed a MITM approach to identify the overlapping features associated with both exposures and outcomes. MITM is a widely used method in high-dimensional settings to identify intermediate biomarkers.^56^ Specifically, we conducted a series of multiple linear regression (i.e., exposure-mediator) models and logistic regression (mediator-outcome) models to evaluate the association of metabolic features with exposures and outcomes, respectively, using the following equations:

1 ln⁡(featurej)=β0j+β1jPM2.5+β2jage+β3jeducation+β4jsex+β5jBMI+β6jMET+β7jtobacoor marijuan use +β8jgestational_age_at_sampling+ϵj

2 $$\log it\left(P(birth)\right)=\theta_{0j}+\theta_{1j}\;\ln\left({feature}_{j}\right)+\theta_{2j}{\mathrm{PM}}_{2.5}+\theta_{3j}age+\theta_{4j}education+\theta_{5j}sex+\theta_{6j}BMI+\theta_{7j}parity+\theta_{8j}tobaco\;or\;marijuana\;use+\theta_{9j}gestational\_age\_at\_sampling$$

where ln (*Feature*) refers to the natural log of intensity of metabolic feature *j*; PM_2.5_ is the averaged PM_2.5_ exposure for a specific window; and MET is the corresponding meteorological covariate; Birth denotes either PTB or ETB with the FTB group as the referent category (i.e., we contrasted PTB and ETB with FTB by two separate models); we included PM_2.5_ in the mediator-outcome model to block the direct effect of PM_2.5_ exposure on the outcome, which may confound the mediator-outcome association. We constructed the two equations following a sophisticated causal mediation framework with adjustment of exposure-mediator confounders and mediator-outcome confounders (Figure S1) in eqs 1 and 2, respectively.^57^ Then, we further focused on the 398 confirmed metabolites with confidence level 1 and applied Benjamini-Hochberg procedure on those features to adjust for multiple comparison correction.^58^ The significance threshold was set at adjusted *p*-values (FDR_B–H_) < 0.2. Results were presented using Manhattan plots (Figures S2 and S3 in the Supporting Information).

To aid the interpretation of the MITM approach and HDMA results, we conducted a pathway enrichment analysis using the R package *metapone* based on the significant metabolic features identified in both MWAS. *Metapone* is a novel bioinformatic platform to predict functional biological activities of untargeted metabolomic data extracted in both positive and negative ESI together, which developed a pathway database combining the Small Molecule Pathway Database (SMPDB) and mummichog database.^59^ The inputs of metabolic features were putatively annotated with the related weights calculated based on the uncertainty in metabolite-feature matching, and then the significance of enriched biological pathways was tested taking into account the weight schema.^59^ The biological pathways associated with either PM_2.5_ exposures or outcomes with more than one metabolite enriched and a *p*-value <0.05 were included for further detecting the overlapping pathway.

As a complementary means to identify the potential mediating features between PM_2.5_ exposures and the gestational age at birth outcome categories, we employed HDMA via the R package *HIMA* (version 2.2.1).^60^ Previous researchers have developed a framework of mediation analysis that is able to deal with multiple mediators simultaneously and tease apart the indirect effect of individual mediator, which was depicted elsewhere in detail.^61^ *HIMA* expands this multiple mediator framework to the high-dimensional setting by reducing the dimensionality of omics data, and the significant mediators were reported with multiple testing correction.^60^ Compared with the aforementioned MITM approach, *HIMA* is able to incorporate multiple mediators in a single mediator-outcome model, which enables us to ascertain the extent to which the indirect effects are explained by the mediators. Separate analysis on the annotated features was conducted for each column (HILIC positive ESI and C18 negative ESI). We selected confounders based on the same criteria used in the meet-in-the-middle approach (Figure S1). *HIMA* allows for specifying distinct confounder sets for the mediator and outcome models, respectively. In the mediator model, we adjusted for maternal age, maternal educational attainment, maternal BMI, infant sex, tobacco and marijuana use, meteorological factors, and gestational age at blood sample collection. The outcome models included adjustments for PM_2.5_ exposure (to block potential backdoor paths), maternal age, maternal educational attainment, infant sex, maternal BMI, tobacco and marijuana use, parity, and gestational age at blood sample collection.

We also performed sensitivity analyses in which we evaluated gestational age at birth in completed weeks as a continuous outcome to conduct HDMA and controlled for the same set of covariates as the main analysis. For this analysis, we considered gestational age at birth in completed weeks from 20 through 39 weeks, assigning 39 weeks to all births that attained at least 39 weeks, as in previous research.^62^ To assess the potential nonlinear influence of meteorological factors, we conducted sensitivity analyses by including natural cubic spline terms (3 degrees of freedom) for temperature and relative humidity in the exposure–mediator models. To test the robustness of the exposure-mediator model to pregnancy complications, we included hypertension disorders during pregnancy and gestational diabetes as additional covariates.

All analyses were completed in *R* (version 4.2).

## Results

A total of 330 individuals from the Atlanta AA cohort were included in the current analysis, and their demographic characteristics are described in Table 1, stratified by gestational age at birth outcome category. Participants with PTB had the lowest early pregnancy BMI, highest proportion of multiparity, highest infant sex ratio (Male vs Female ≈ 3:2), and highest proportion of maternal tobacco and marijuana use; while those with ETB had the lowest proportion of multiparity, highest proportion of maternal alcohol use, and other covariates with a similar distribution compared to those with an FTB. We did not observe a significant difference in gestational age at biosampling among the three groups. Within the full-term group, 88 participants (26.7%) had a gestational age >40 weeks, and the maximum gestational age in the study population was 41.6 weeks.

**Table 1:** Characteristics of Analytic Sample of Participants Enrolled in Atlanta African American Maternal-Child Cohort Study, 2014–2018 (*N* = 330), by Gestational Age at Birth Outcome Category^a^

	preterm (*N* = 66)	early term (*N* = 54)	full term (*N* = 210)	*p*
maternal age, years				
≤20	15 (22.7)	8 (14.8)	40 (19.0)	0.766
>20 & ≤30	43 (65.2)	36 (66.7)	135 (64.3)	
>30	8 (12.1)	10 (18.5)	35 (16.7)	
maternal educational attainment, no. (%)				
less than high school	12 (18.2)	14 (25.9)	28 (13.3)	0.044
high school	30 (45.5)	20 (37.0)	73 (34.8)	
some college or more	24 (36.4)	20 (37.0)	109 (51.9)	
body mass index, mean (SD)	27.1 (6.91)	28.1 (8.17)	29.2 (7.68)	0.154
infant sex, no. (%)				
male	40 (60.6)	26 (48.1)	98 (46.7)	0.134
female	26 (39.4)	28 (51.9)	112 (53.3)	
parity, no. (%)				
nulliparity	28 (42.4)	16 (29.6)	104 (49.5)	0.013
primiparity	13 (19.7)	22 (40.7)	55 (26.2)	
multiparity	25 (37.9)	16 (29.6)	51 (24.3)	
maternal tobacco or marijuana use, no. (%)				
no	36 (54.5)	33 (61.1)	119 (56.7)	0.778
yes	30 (45.5)	21 (38.9)	91 (43.3)	
gestational age at blood draw, weeks, mean (SD)	11.6 (2.29)	11.6 (2.30)	11.4 (2.14)	0.600
gestational age at birth, weeks, mean (SD)	33.6 (3.94)	37.7 (0.60)	39.9 (0.70)	<0.001
season of conception, no. (%)				
spring (March–May)	20 (30.3)	12 (22.2)	52 (24.8)	0.894
summer (June–Aug)	21 (31.8)	18 (33.3)	67 (31.9)	
fall (Sept–Nov)	15 (22.7)	12 (22.2)	45 (21.4)	
winter (Dec–Feb)	10 (15.2)	12 (22.2)	46 (21.9)	
apparent temperature, mean (SD)				
first trimester	67.5 (12.3)	64.8 (12.2)	65.8 (13.1)	0.497
one week prior to blood draw	65.2 (16.2)	61.5 (14.3)	65.3 (14.5)	0.247
one month prior to blood draw	66.8 (14.9)	62.7 (13.0)	65.5 (14.1)	0.266

a Abbreviations: SD, standard deviation; min, minimum; max, maximum.

The median of PM_2.5_ exposure during the one-year prior to conception, first trimester, one-week, and one-month prior to blood draw were 9.27 [interquartile range (IQR) = 0.93], 9.02 (2.05), 8.59 (3.25), and 8.88 (2.48) μg/m^3^, respectively (Table S2). The long-term exposure (i.e., one-year average) was weakly to moderately correlated with the three short-term exposures, whereas the short-term exposures were moderately to strongly correlated with each other (Figure S4A). Participants living in Downtown and Midtown Atlanta neighborhoods, where several highways intersect, had a higher level of one-year exposure compared to those in areas located on the outskirts of the city. We did not observe the same tendency for the short-term exposure (Figure S4B), which is expected, as short-term exposure to PM_2.5_ is more likely affected by seasonal variability.^63^

### Metabolome-Wide Association Analysis

We analyzed 11,269 and 9565 metabolic features for the HILIC and C18 columns, respectively. There were 164, 73, 8, and 135 metabolic features associated (adjusted *p*-value FDR_B–H_ <0.2) with PM_2.5_ exposures during one year prior to conception, first trimester, one week prior to blood draw, and one month prior to blood draw, respectively (Table S3). When focusing on the 398 metabolites confirmed with Level 1 evidence, we identified three metabolites significantly associated with one-year exposure prior to conception, one metabolite with first trimester exposure, one metabolite with exposure during one-week prior to blood draw, and three metabolites with exposure during one-month prior to blood draw in the HILIC column (FDR_B–H_ <0.2). We found two metabolites associated with one-year exposure prior to conception in the C18 column (FDR_B–H_ <0.2). The detailed statistics of significant metabolic features were summarized in the Supporting Information (Table S4). As shown in Figure 2, the five metabolites associated with one-year exposure prior to conception were hydroxypyridine, quinoline, carnitine, acetylcysteine, and adenosine triphosphate (ATP); Phthalic anhydride was associated with both first trimester exposure and one-month exposure prior to blood draw; Adenosine was associated with one-week exposure prior to blood draw; Di­(2-ethylhexyl)­phthalate (DEHP) and lysope(20:3) were associated with one-month exposure prior to blood draw.

**Figure 2:**
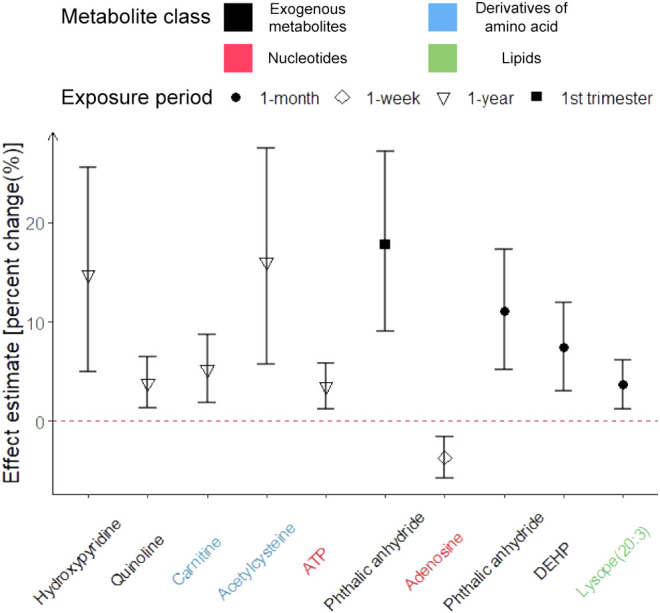
Confirmed metabolites associated with periconceptional exposures to ambient fine particulate matter (PM_2.5_) among pregnant participants in the Atlanta African American Maternal-Child Cohort, 2014–2018. The effect estimate is associated with one-unit (μg/m^3^) increase in PM_2.5_ exposures. We log-transformed the relative intensity of metabolites, so the effect estimate was denoted as percent change [%, (*e* ^β^–1) × 100]. The exogenous metabolites were marked in black, derivatives of amino acids in blue, nucleotides in red, and lipids in green. Metabolites shown met the significance threshold of FDR_B–H_ < 0.2, based on Benjamini-Hochberg adjusted *p*-values.

The number of untargeted metabolic features associated with early birth is summarized in Table S3. Among the Level 1 confirmed metabolites, 20 metabolites were significantly associated with the risk of ETB, and three were associated with PTB (FDR_B–H_ < 0.2). The detailed statistics of significant features are summarized in the Supporting Information (Table S5). Among these metabolites, the associations of 12 metabolites with the risk of ETB were negative and independent of all PM_2.5_ exposure windows, including alanine, choline, proline, hydroxyproline, creatine, leucine, histidine, citrulline, serotonin, tyrosine, cystine, and cortexolone. Carnitine was associated with a lower risk of ETB birth, independent of first trimester and one-month PM_2.5_ exposure prior to blood draw.

We used the *R* package *metapone* to identify the biological pathways enriched by metabolic features associated with PM_2.5_ exposures (Table S6) and early birth outcomes (Table S7). Eight pathways were associated with one-year exposure prior to conception, including estrone metabolism; phenylalanine, tyrosine, and tryptophan biosynthesis; purine metabolism; serotonergic synapses; arachidonic acid metabolism; TCA cycle; and tryptophan metabolism. The first trimester of exposure was associated with retinol metabolism. Four pathways were associated with 1 week of exposure prior to blood draw, including phenylalanine metabolism, protein digestion and absorption, tryptophan metabolism, and biopterin metabolism. No pathway was found to be associated with a one-month exposure. In contrast, more biological pathways were identified in relation to early birth outcomes: five distinct pathways were associated with preterm birth (PTB) and 32 distinct pathways were associated with early term birth (ETB) (Table S7).

Sensitivity analyses including hypertensive disorders of pregnancy and gestational diabetes as covariates in the exposure–mediator models yielded results consistent with our main findings. The number of significant metabolic features (FDR_B–H_ <0.2) associated with PM_2.5_ exposure remained largely unchanged (Table S8), indicating that these maternal complications did not substantially confound the observed associations. Sensitivity analyses incorporating natural splines for temperature and humidity yielded similar results, with a consistent number and identity of PM_2.5_-associated metabolic features observed (Table S9), indicating that the main findings are robust to alternative specifications of meteorological covariates.

### Meet-in-the-Middle Approach

Following the MITM framework, various unique metabolic features (*N* ranging from 34 to 113) were identified as overlapping metabolites between PM_2.5_ exposure and ETB or PTB (*p*-values <0.05, Table S10). However, no metabolites were deemed significant after multiple comparison correction. To identify biological pathways potentially mediating the association of PM_2.5_ exposures with PTB and ETB, we characterized the overlapping biological pathways enriched by all annotated and unannotated significant metabolic features at *p*-value <0.05 using *metapone*. Two biological pathways were found to potentially mediate the associations between PM_2.5_ exposure and early birth (Figure 3), including phenylalanine, tyrosine, and tryptophan biosynthesis, which was the overlapping pathway between one-year exposure prior to conception, one-week exposure prior to blood draw, and ETB, and protein digestion and absorption pathway, which was associated with one-week PM_2.5_ exposure and both PTB and ETB. In the phenylalanine, tyrosine, and tryptophan biosynthesis, four overlapping metabolites were identified, including phosphoenolpyruvic acid, tryptophan, phenylpyruvic acid, and indole (Table S11). In the protein digestion and absorption pathway, six overlapping metabolites were identified, including tryptophan, tyrosine, leucine, valine, isoleucine, and piperidine (Table S11). It is important to note that these metabolic features were annotated by *metapone* with a low confidence level of 4, and therefore, the results should be interpreted with caution.

**Figure 3:**
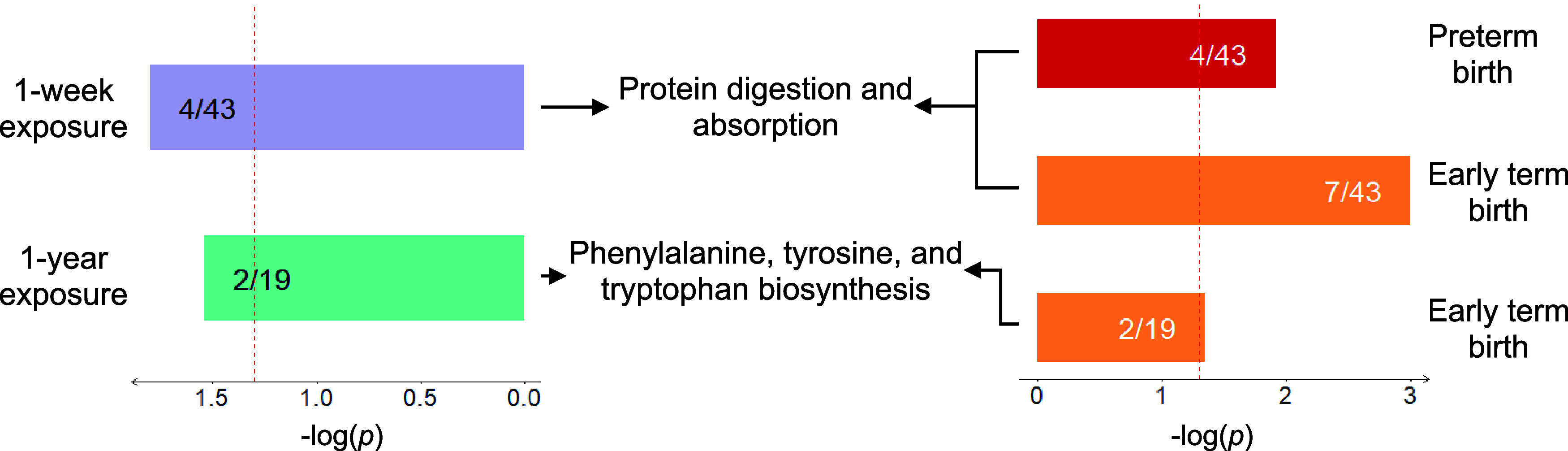
Overlapping biological pathways detected by the meet-in-the-middle approach coupled with the pathway enrichment analysis. Each significant biological pathways were enriched by at least two significant metabolic features associated with either PM_2.5_ exposure or early birth. The number of enriched significant metabolic features (a) and the number of total metabolites in the corresponding pathway (b) were labeled as “a/b” on each bar.

### High-Dimensional Mediation Analysis

Eight confirmed metabolites were identified using HDMA at the significant level of adjusted FDR_B–H_ < 0.2 (Figure 4), among which two had a positive indirect effect estimate (Table S12). Cortexolone mediated a positive association between 1 week of exposure prior to blood draw and the risks of PTB and ETB. Lysope(20:3) mediated a positive association between one month of exposure prior to blood draw and the risk of PTB. Proline and cortexolone were found by the MWAS analysis to be negatively associated with ETB as well (Table S4), while lysope(20:3) was positively associated with one-month PM_2.5_ exposure (Figure 2).

**Figure 4:**
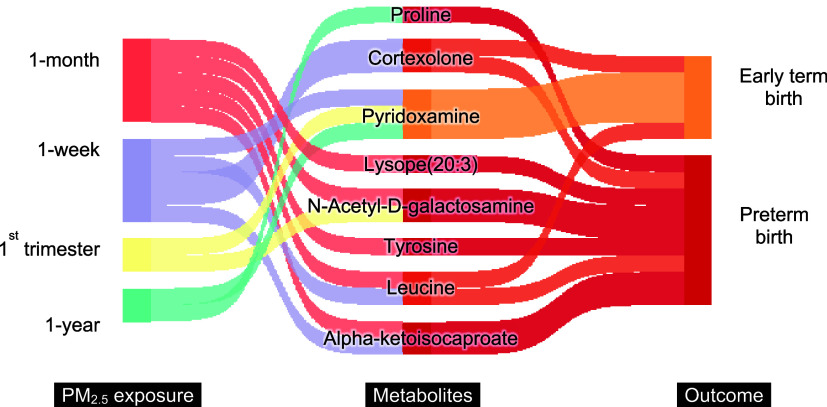
Significant metabolic features (adjusted *p*-value <0.2 via Benjamini-Hochberg procedure) mediating PM_2.5_ exposure and early birth detected by high-dimensional mediation analysis. For ease of reading, the links from exposure to the mediator and from the mediator to outcomes have been color-coded based on PM_2.5_ exposure periods or early birth outcomes. The size is proportional to the number of mediated indirect associations.

In the sensitivity analysis evaluating the outcome of gestational age at birth continuously (in completed weeks) in HDMA, we did not observe any significant annotated metabolic features associated with PM_2.5_ exposure at FDR_B–H_ < 0.2.

## Discussion

To the best of our knowledge, this is the first study investigating the metabolic signatures underlying the association between prenatal exposure to ambient PM_2.5_ and early birth (PTB and ETB). Using a novel parallel strategy combining the MITM and HDMA approaches, we identified two important metabolic pathways, the protein digestion and absorption as well as the aromatic amino acid (phenylalanine, tyrosine, and tryptophan) biosynthesis, which potentially mediated the associations of both long- and short-term exposures to PM_2.5_ with PTB and ETB. Further, we identified cortexolone, a metabolic intermediate in the synthesis of cortisol, mediated a positive indirect effect between PM_2.5_ exposure during one week prior to blood draw and early birth risk, as well as lysope(20:3), which mediated a positive association between one-month exposure prior to blood draw and the PTB risk. Additionally, we uncovered multiple novel endogenous metabolites, including carnitine, adenosine, lysope (20:3), acetylcysteine, and ATP, associated with PM_2.5_ exposure, with ten essential and nonessential amino acids associated with elevated risk of PTB and ETB. Together, these findings provide novel insights supporting the role of maternal metabolomics perturbations in mediating the associations between prenatal PM_2.5_ exposure and early birth outcomes in this cohort of AA individuals.

A growing body of previous studies has characterized the maternal metabolome in response to air pollution exposure during pregnancy and/or the preconception time period.^64–70^ However, only two studies, including one of our previous publications, focus on examining the metabolic intermediates of air pollution and early birth-related outcomes.^31,65^ Our previous publication on the same AA Maternal-Child Cohort found that urea cycle/amino group metabolism in early pregnancy was associated with both total cotinine concentration in urine samples and the risk of preterm birth, while no overlapping metabolites were identified.^31^ Additionally, Zheng et al. reported that hydrogen phosphate levels in early pregnancy may mediate the association between PM_2.5_ exposure in the first trimester and PTB risk, suggesting the involvement of oxidative phosphorylation pathway.^65^ To address this critical gap, we employed an innovative parallel strategy by applying both the MITM approach and HDMA to identify the metabolomic signatures and pathways associated with both PM_2.5_ exposure and the risk of PTB and ETB. Using the MITM approach, we did not find any overlapping metabolic features associated with both PM_2.5_ exposure and early birth outcomes after multiple comparison correction. This null finding is likely due to the limited statistical power in the context of a relatively modest sample size, compounded by the conservative nature of multiple comparison correction in high-dimensional metabolomics data set. Nevertheless, the MITM approach, complemented by pathway enrichment analyses, identified several biologically plausible pathways that may mediate the effects of PM_2.5_ on early birth risks, providing valuable hypothesis-generating insights for future studies. In HDMA, the most notable finding is the identification of two novel metabolites mediating the positive association between PM_2.5_ and the risk of PTB and ETB. Specifically, we uncovered cortexolone, also known as 11-deoxycortisol, an endogenous glucocorticoid steroid and a metabolic intermediate in the synthesis of cortisol which is a potential contributor to premature labor.^71^ During pregnancy, the placenta produces corticotropin-releasing hormone (CRH), which enters maternal circulation and stimulates the production of cortisol.^71^ In turn, elevated circulating cortisol levels further upregulate placenta CRH production, creating a positive feedforward loop that accelerates labor processes.^71^ Cortisol also promotes prostaglandin production and cervical ripening, both essential for labor onset.^71^ As a key stress hormone, persistently high cortisol levels—due to environmental or psychosocial stress, may dysregulate the maternal stress response and increase the risk of preterm birth. The existing studies on air pollution and levels of cortexolone is limited with mixed findings, where an increased serum level of cortexolone was observed among male Sprague–Dawley rats exposed to carbon black nanoparticles for 90 days,^72^ while no significant association was observed between long-term residential exposure to PM_2.5_ and serum cortexolone level in a population-based cohort study of 6670 Chinese rural residents aged 18–79 years.^73^ Meanwhile, a previous study has reported increase in the level of cortexolone in the early third trimester among pregnant people with spontaneous PTB < 32 weeks compared to those with spontaneous PTB ≥ 32 weeks.^74^ In our study, elevated short-term PM_2.5_ exposure was associated with decreased intensities of cortexolone in the maternal metabolome, which in turn, was associated with increased risks of both PTB and ETB among pregnant African Americans, revealing a potential important role of cortexolone in mediating the effects of PM_2.5_ exposure on early births, which warrants further investigation.

Another novel intermediate metabolite showing positive mediation effects is lysoPE(20:3), a member of the lysophospholipid family that plays a key role in cell membrane dynamics, signaling, and inflammatory responses. Few evidence can be found between lysoPE(20:3) and air pollution exposure in the existing literature. However, lysophospholipids, including lysoPEs, are bioactive lipids that influence processes such as immune cell activation, oxidative stress, and vascular function,^75^ all of which are hallmarks and can be disrupted by PM_2.5_ exposure. Meanwhile, lysoPEs are essential for maintaining cell membrane integrity, supporting fetal development, and modulating immune responses during pregnancy, alterations in which have been linked to adverse pregnancy outcomes, including PTB. Using HDMA, we observed a significant positive mediating effect of lysoPEs, where increased levels of PM_2.5_ exposure was associated with increased intensities of lysoPEs, which resulted in increased odds of PTB among our study participants. More research on lysope(20:3) is warranted to investigate its role as both a potential biomarker and a mechanistic link between PM_2.5_ exposure and early birth outcomes.

Using MITM, we identified two important biological pathways associated with both PM_2.5_ exposure and the risk of PTB and ETB. Specifically, the protein digestion and absorption metabolic pathway plays a critical role in breaking down dietary proteins into amino acids and small peptides, which are then absorbed in the small intestine and utilized for various physiological functions, including energy production, tissue repair, and cellular signaling.^76^ Upon elevated exposures to PM_2.5_ during pregnancy, disruptions in this pathway may lead to an imbalance in maternal and fetal nutrient supply and amino acid availability, significantly impacting systemic metabolic and inflammatory responses,^76^ ultimately raising the risks of PTB and ETB. Meanwhile, the phenylalanine, tyrosine, and tryptophan metabolism plays a critical role in producing key biomolecules such as neurotransmitters, hormones, and metabolites involved in immune regulation and oxidative stress.^77^ PM_2.5_ exposure has been linked with perturbations in this pathway by triggering inflammation, oxidative stress, and shifts in metabolite production,^18,29^ which may disrupt maternal-fetal signaling and stress responses. This pathway is highly relevant to PTB and ETB, as disturbances in these metabolites are linked to inflammation, immune dysregulation, and impaired placental function, all of which are critical contributors to early birth outcomes. Meanwhile, we identified various intermediate metabolites in MITM, though few passed the multiple testing corrections, possibly due to the limited statistical power. Nevertheless, many of these metabolites have been consistently reported in previous metabolomics investigations on either air pollution exposure or adverse birth outcomes. For instance, we identified carnitine, a cofactor critical for fat metabolism, which has also been reported in these air pollution metabolomics studies.^18^ Consistently, we identified carnitine in the current investigation, which was positively associated with long-term exposure to PM_2.5_. Carnitine actively participates in mitochondrial processes by facilitating the transport of fatty acids into the mitochondria and regulating the Coenzyme A (CoA)/acylCoA ratio within the mitochondria, which resulted in an essential role in energy metabolism, particularly in tissues that heavily rely on fatty acid oxidation for energy production.^78^ Given that we observed associations between adenosine and ATP and first trimester exposure and one-year exposure, respectively, the present findings suggested that periconceptional PM_2.5_ exposure might disrupt energy metabolism during pregnancy. Taking these findings together, we hypothesized a potential molecular network (Figure 5) in which energy metabolism and amino acid metabolism might work together to mediate the association between PM_2.5_ exposure and the risks of PTB and ETB.

**Figure 5:**
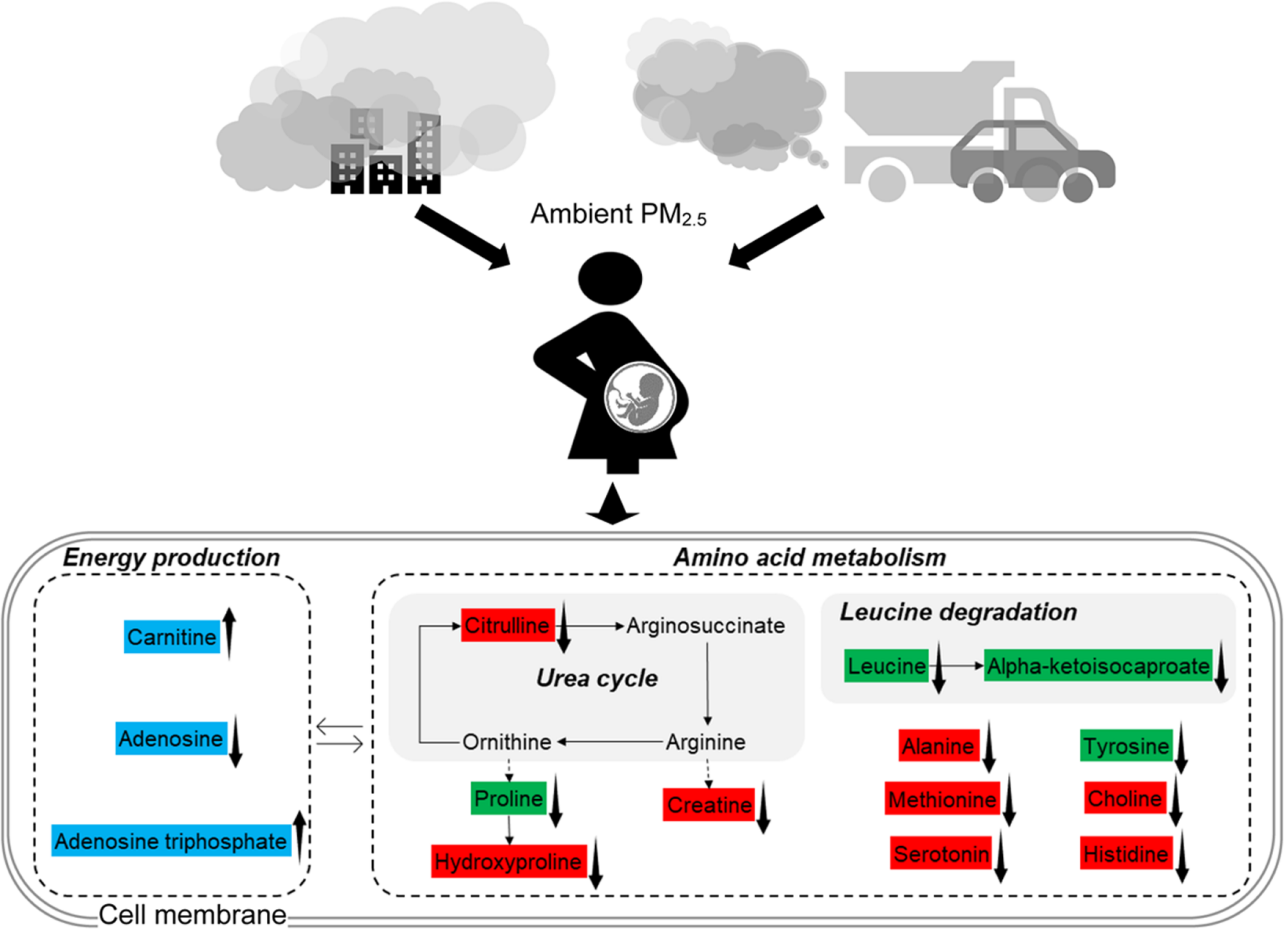
Potential molecular mechanisms partially illustrated as metabolic networks for the association between PM_2.5_ exposure and early birth among pregnant participants in the Atlanta African American Maternal-Child Cohort, 2014–2018. We colored metabolites associated with PM_2.5_ in blue, metabolites associated with PTB or ETB in red, and metabolites mediating the association in green. Dashed arrows denote the multiple reactions required. Figure was created by the authors using Microsoft PowerPoint.

This discrepancy in the findings between MITM and HDMA likely reflects differences in their methodological frameworks. MITM identifies mediators by separately testing associations between exposure and metabolic features and between metabolic features and outcomes, with multiple testing corrections applied at both stages. This approach assumes independence among features, which may limit statistical power in the presence of correlated metabolic signals. In contrast, HDMA evaluates all candidate metabolic mediators simultaneously into a unified high-dimensional model, leveraging covariance among features to enhance the power and improve detection of potential mediators. As such, the two methods offer complementary insights, with HDMA providing greater sensitivity in this highly dimensional metabolomics context.

We also conducted the sensitivity analysis using continuous gestational age at birth as the outcome in HDMA. Possibly due to the relatively small effect size of PM_2.5_ on gestational age at birth, we did not identify significant metabolic intermediates, consistent with the main analysis. This may also be explained by the potential nonlinear relationship between PM_2.5_ exposure and gestational age at birth, where Qiu et al. previously found that newborns with lower gestational age at birth were at a higher risk of gestational age reduction associated with PM_2.5_ exposure during the third trimester using quantile regression.^79^ Thus, our use of categorical birth outcomes in the main analysis may better evaluate the effects of PM_2.5_ exposure on maternal metabolome and risks of early births, which provides a clear interpretation in clinical settings.

Despite these promising findings, we identified several limitations and key areas for future work. Given the study design, our results may not necessarily imply a causal relationship. All serum samples were collected in early and midpregnancy such that we were not able to examine metabolomic profiles in the potentially critical exposure windows in later pregnancy. The sampling date of blood spanned from 6 to 17 gestational weeks in the current analysis, and the PM_2.5_ exposure assessed for the first trimester may not completely precede the measure of metabolic profiles for some participants. To address this pitfall, we examined two exposure windows relevant to the sampling date (i.e., one week and one month prior to blood draw). While we characterized both long-term and short-term PM_2.5_ exposure, the associations between one year of exposure prior to conception and metabolic profiles measured in early pregnancy should be interpreted with caution. Metabolite levels are dynamic and can be influenced by short-term changes such as diet,^89^ physical activity,^90^ and maternal stress,^91^ especially during pregnancy. However, prior studies by our group and others have demonstrated that certain metabolomic signatures can persist and reflect longer-term environmental exposures, including ambient air pollution. These findings are supported by consistent evidence from independent cohorts and systematic reviews.^18,24,32,43,44,64,92,93^ Nevertheless, the temporal mismatch between long-term exposure windows and single-point metabolomic sampling may introduce uncertainty, and future studies incorporating repeated biospecimen collection (i.e., longitudinal biomonitoring at early, middle, and late pregnancy) are warranted to better capture the exposure-related metabolic changes across various critical time windows. Additionally, we estimated ambient PM_2.5_ exposure based on the residential address of the cohort participants, which could be underestimated due to occupational mobility and commuting mode of pregnant women.^81^ This source of exposure measurement error may be nondifferential, which compromised the power of analysis. In this high-dimensional hypothesis-generating study, we used adjusted *p*-values (FDR_B–H_) <0.2 as the significance threshold to balance discovery with false positive control. While we acknowledge that this more lenient threshold may increase the risk of false discoveries, it also helps reduce the likelihood of false negatives—i.e., missing true associations due to overly stringent multiple testing correction. This approach is commonly adopted in air pollution metabolomics studies to facilitate signal detection.^18^ Nonetheless, further validation in larger and independent cohorts is warranted. Selection bias may have occurred, as enrollment was limited to individuals who sought prenatal care and voluntarily participated in the study. As a result, our findings may not fully reflect associations among populations with limited access to healthcare or lower research participation. However, the observed PTB rate (20%) in our study population was comparable to the state-level estimates,^83,84^ indicating that any potential selection bias was likely minimal. Finally, this study focused on PM_2.5_ as the primary exposure of interest due to its well-established links to preterm birth and its suitability for integration with untargeted metabolomic data. Although other gaseous pollutants such as NO_2_ and O_3_ may also play a role in early birth risk or act as coexposures, we did not include them in the current analysis because reliable exposure estimates for these pollutants were not available at the time of study execution. Additionally, including these correlated pollutants could introduce multicollinearity,^85^ complicating the interpretation of exposure-omics relationships in this high-dimensional setting. Future studies incorporating multipollutant frameworks or advanced mixture analysis techniques are warranted to elucidate the combined effects of co-occurring air pollutants.^23,86–88^

Our study also has several notable strengths. First, we employed an innovative parallel and complementary strategy using both the MITM and HDMA approaches. The MITM approach could identify potential intermediate factors by targeting overlapping metabolic features in potential biological pathway connecting PM_2.5_ exposure to PTB or ETB. This would aid posterior identification of overlapping biological pathways and facilitate biological interpretation of the untargeted metabolomic data. HDMA incorporates multiple metabolic features into a single mediator-outcome model, which teases apart the complex indirect effect of PM_2.5_ exposure on PTB and ETB for each metabolic mediator. Although we applied two complementary analytical approaches (MITM and HDMA) to identify metabolomic mediators, our findings should be interpreted as hypothesis-generating and require validation in independent birth cohorts using alternative statistical frameworks. Second, the gestational age at birth was well characterized in our cohort based on the early pregnancy dating and ascertained following ACOG guidelines.^33^ Specifically, medical personnel documented the gestational age clearly in the medical record based on the last menstrual period and early pregnancy ultrasound examination, which minimized the misclassification bias of outcomes.^33^ Third, our study participants were exclusively AA, a population that has been largely under-represented in environmental epidemiologic studies, despite bearing the “double jeopardy” of multiple environmental stressors and elevated adverse birth outcomes. While the findings from this study may not directly generalize to other population, they offer important insights into persistent health burden of PTB and ETB among African American populations.^94^ Future studies comparing metabolomics results across diverse populations could further elucidate differences in biological susceptibility and environmental responses. Last, our workflow of the high-resolution metabolomics profiling was well established and has been shown to successfully analyze many nonfasting samples previously.^32,95^

In conclusion, using high-resolution metabolomics, we identified various novel metabolic signatures and pathways associated with long- and short-term periconceptional PM_2.5_ exposure in a well-established prospective AA birth cohort. Specifically, based on an innovative parallel strategy of utilizing MITM and HDMA, our findings point to the critical roles of energy metabolism and amino acid metabolism in mediating the association of PM_2.5_ exposure with the risks of PTB and ETB in our understudied population of AA pregnant individuals. Our findings also provide important information about the metabolic perturbations that appear to mediate the association between PM_2.5_ and early birth outcomes (PTB and ETB). Notably, one observed mediator, cortexolone, indicates the potential role of endogenous steroid hormones in regulating the adverse effects of PM_2.5_ on early births. Together, these findings contribute to our understanding of the biological mechanisms underlying PM_2.5_ toxicity in pregnancy. The identification of specific metabolic signatures and pathways highlights the importance of integrating environmental health considerations into clinical practice. These insights may help characterize PM_2.5_-related susceptibility to PTB and ETB and potentially inform the development of metabolomics-based screening tools or interventions aimed at mitigating PM_2.5_-induced health risks.

## Supplementary Material



## Data Availability

The raw and processed metabolomics data generated in this study have been deposited in the Metabolomics Workbench (https://www.metabolomicsworkbench.org/ Study ID ST002692). The clinical outcome data are available under restricted access to protect the privacy of the study participants; access can be obtained by emailing Drs. D.L. and A.L.D. Requests will be addressed within 10 business days. The demographic covariates data are protected and are not available due to data privacy laws.

## Notes

DAGs, Manhattan plots, correlation maps, summary statistics of exposures and metabolites, detailed MWAS and mediation results, pathway enrichment outputs, sensitivity analyses, and metabolite IDs mapped to enriched pathways (PDF)
