# Supplementary material for: Unraveling the Molecular Links between Fine Particulate Matter Exposure and Early Birth Risks in African American Mothers: A Metabolomics Study in the Atlanta African American Maternal-Child Cohort
Source: Environ Sci Technol. 2025 May 29;59(22):10905–18. doi: 10.1021/acs.est.5c02071 (PMC12164266; doi:10.1021/acs.est.5c02071)
Supplement: Supplementary file 1 [file es5c02071_si_001.pdf]

Supporting Information

Unraveling the Molecular Links Between Fine Particulate Matter Exposure and Early Birth Risks in African American Mothers: A Metabolomics Study in the Atlanta African American Maternal-Child Cohort

Authors: Zhenjiang Li[1*], Anne L. Dunlop[2], Jeremy A. Sarnat[1], Anke Hüls[3,1,4], Stephanie M. Eick[1], Audrey Gaskins[1,3], Howard Chang[1,4], Armistead Russell[5], Youran Tan[1], Haoran Cheng[1], Dana Boyd Barr[1], Alicia K. Smith[2], Carmen Marsit[1], Dean P. Jones[6], Donghai Liang[1,3*]

Affiliations:
[1] Gangarosa Department of Environmental Health, Rollins School of Public Health, Emory University; Atlanta, GA 30322, USA
[2] Department of Gynecology and Obstetrics, School of Medicine, Emory University; Atlanta, GA 30322, USA
[3] Department of Epidemiology, Rollins School of Public Health, Emory University; Atlanta, GA 30322, USA
[4] Department of Biostatistics, Rollins School of Public Health, Emory University; Atlanta, GA 30322, USA
[5] School of Civil and Environmental Engineering, Georgia Institute of Technology; Atlanta, GA 30332, USA
[6] Department of Medicine, School of Medicine, Emory University; Atlanta, GA 30322, USA

[*] Corresponding author: Zhenjiang Li, PhD, and Donghai Liang, PhD, Gangarosa Department of Environmental Health, Rollins School of Public Health, Emory University, 1518 Clifton Rd NE, Atlanta, GA 30322, USA. Tel: 404-712-9583, Email: zhenjiang.li@emory.edu, donghai.liang@emory.edu

Contents of Supporting Information

Total pages: 21

Figures: 4 (Figures S1-S4)

Tables: 12 (Tables S1-S12)

Figure S1. Directed acyclic graph of the confounding structure.
Figure S2. Manhattan plots of metabolome-wide association analysis in the HILIC column.
Figure S3. Manhattan plots of metabolome-wide association analysis in the C18 column.
Figure S4. The Pearson's correlations among fine particulate matter ($PM_{2.5}$) exposures for the four exposure windows and the spatial variability of the $PM_{2.5}$ illustrated in Metropolitan Atlanta.
Table S1. Distribution of exposures to ambient fine particulate matter ($PM_{2.5}$, μg/m^3) in the four exposure windows and early birth outcomes by maternal marital status among 330 pregnant participants in the Atlanta African American Maternal-Child Cohort, 2014–2018.
Table S2. Distribution of exposure to ambient fine particulate matter ($PM_{2.5}$) in the four exposure windows among 330 pregnant participants in the Atlanta African American Maternal-Child Cohort, 2014–2018.
Table S3. Number of metabolic features associated with $PM_{2.5}$ exposures or early birth outcomes (preterm or early term birth) detected via the MWAS analysis among 330 pregnant participants in the Atlanta African American Maternal-Child Cohort, 2014–2018.

Table S4. Model statistics of significant annotated metabolic features associated (adjusted $p < 0.2$) with ambient $PM_{2.5}$ exposures of different time windows among 330 pregnant participants in the Atlanta African American Maternal-Child Cohort, 2014–2018.
Table S5. Model statistics of significant annotated metabolic features associated (adjusted $p < 0.2$) with early term or preterm birth among 330 pregnant participants in the Atlanta African American Maternal-Child Cohort, 2014–2018.
Table S6. Biological pathways associated with $PM_{2.5}$ exposures detected by the pathway enrichment analysis.
Table S7. Biological pathways associated with early birth outcomes [i.e., preterm birth (PTB) and early-term birth (ETB)] detected by the pathway enrichment analysis, with adjusting for $PM_{2.5}$ exposures.
Table S8. Number of metabolic features associated with $PM_{2.5}$ exposures (FDRB-H < 0.2) with or without adjusting for hypertension disorders of pregnancy and gestational diabetes, detected via the MWAS analysis among 330 pregnant participants in the Atlanta African American Maternal-Child Cohort, 2014–2018.
Table S9. Number of metabolic features associated with $PM_{2.5}$ exposures (FDRB-H < 0.2) with adjusting for apparent temperature or natural smooth function of relative humidity and temperature, detected via the MWAS analysis among 330 pregnant participants in the Atlanta African American Maternal-Child Cohort, 2014–2018.
Table S10. Number of overlapping metabolic features associated $PM_{2.5}$ exposures and early birth, detected via the MWAS analysis among 330 pregnant participants in the Atlanta African American Maternal-Child Cohort, 2014–2018.
Table S11. The significant metabolites mapped in the overlapping biological pathways associated with $PM_{2.5}$ exposures and early birth outcomes detected by the pathway enrichment analysis.
Table S12. Model statistics of the significant mediating metabolites (adjusted p-value < 0.2 via Benjamini-Hochberg procedure) between $PM_{2.5}$ exposure and early birth detected by high-dimensional mediation analysis.

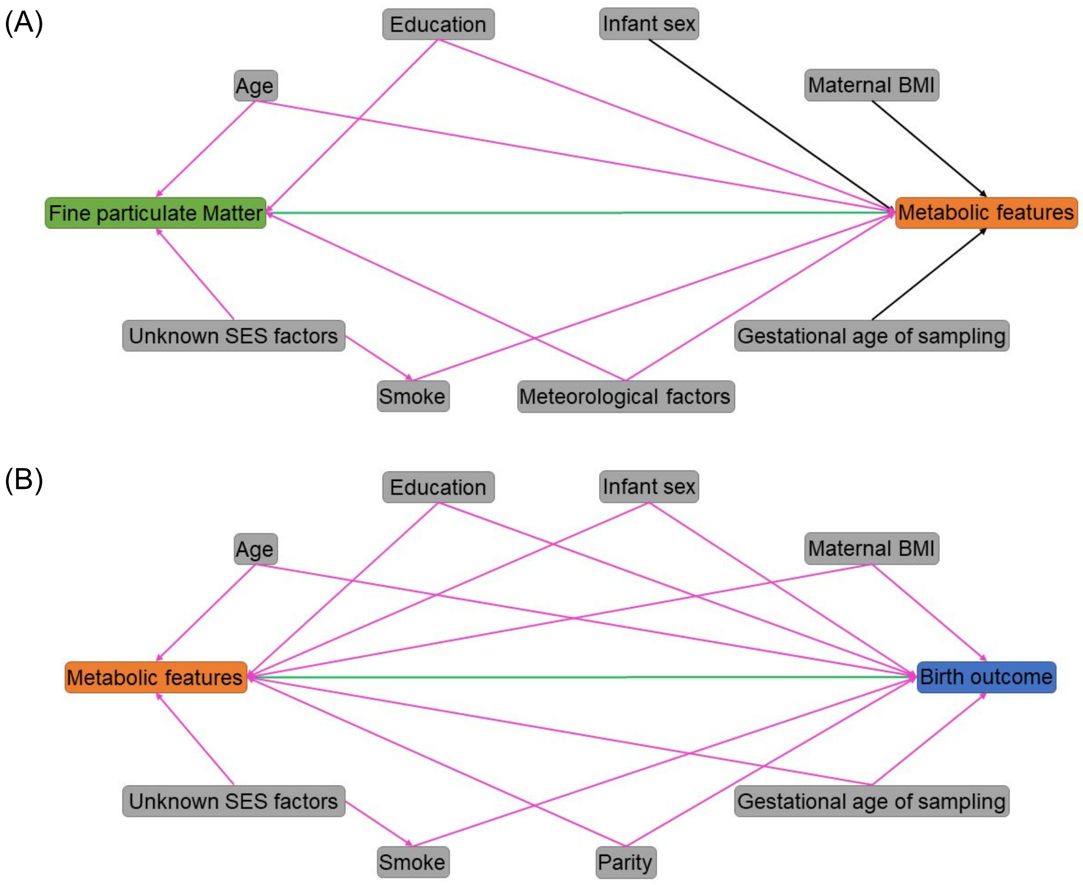

Figure S1. Directed acyclic graph of the confounding structure. (A) The association between fine particulate matter exposure and metabolic features detected in plasma samples. (B) The association between metabolic features and preterm and early term birth. The green line denotes a hypothesized causal path, and the red line denotes a potential biasing path. BMI, body mass index; SES, socio-economic status; Smoke, tobacco and marijuana use in the month prior to pregnancy; Meteorological factors, conception season or averaged apparent temperature.

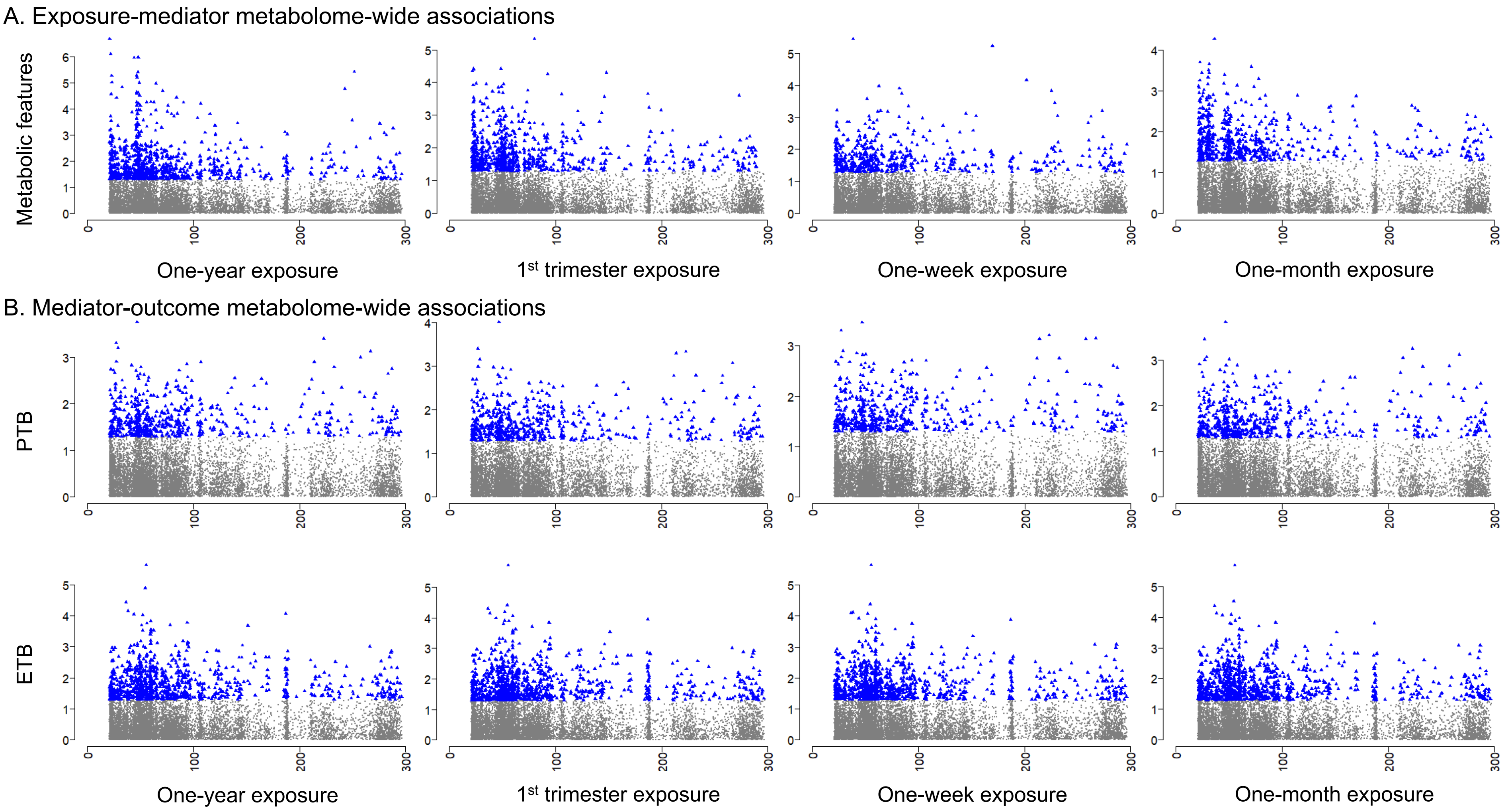

Figure S2. Manhattan plots of metabolome-wide association analysis in the HILIC column. A. Associations between $PM_{2.5}$ exposures and changes in intensities of metabolic features; B. Associations between changes in intensities of metabolic features and PTB or ETB, with adjustment of $PM_{2.5}$ exposures for different time windows separately. X-axis denotes the retention time (in seconds) of the metabolic features, and Y-axis denotes the negative $\log_{10}$ of *p*-values. Blue indicated associations at raw *p*-values < 0.05. Abbreviations: HILIC, hydrophilic interaction liquid chromatography; $PM_{2.5}$, fine particulate matter; PTB, preterm birth; ETB, early term birth.

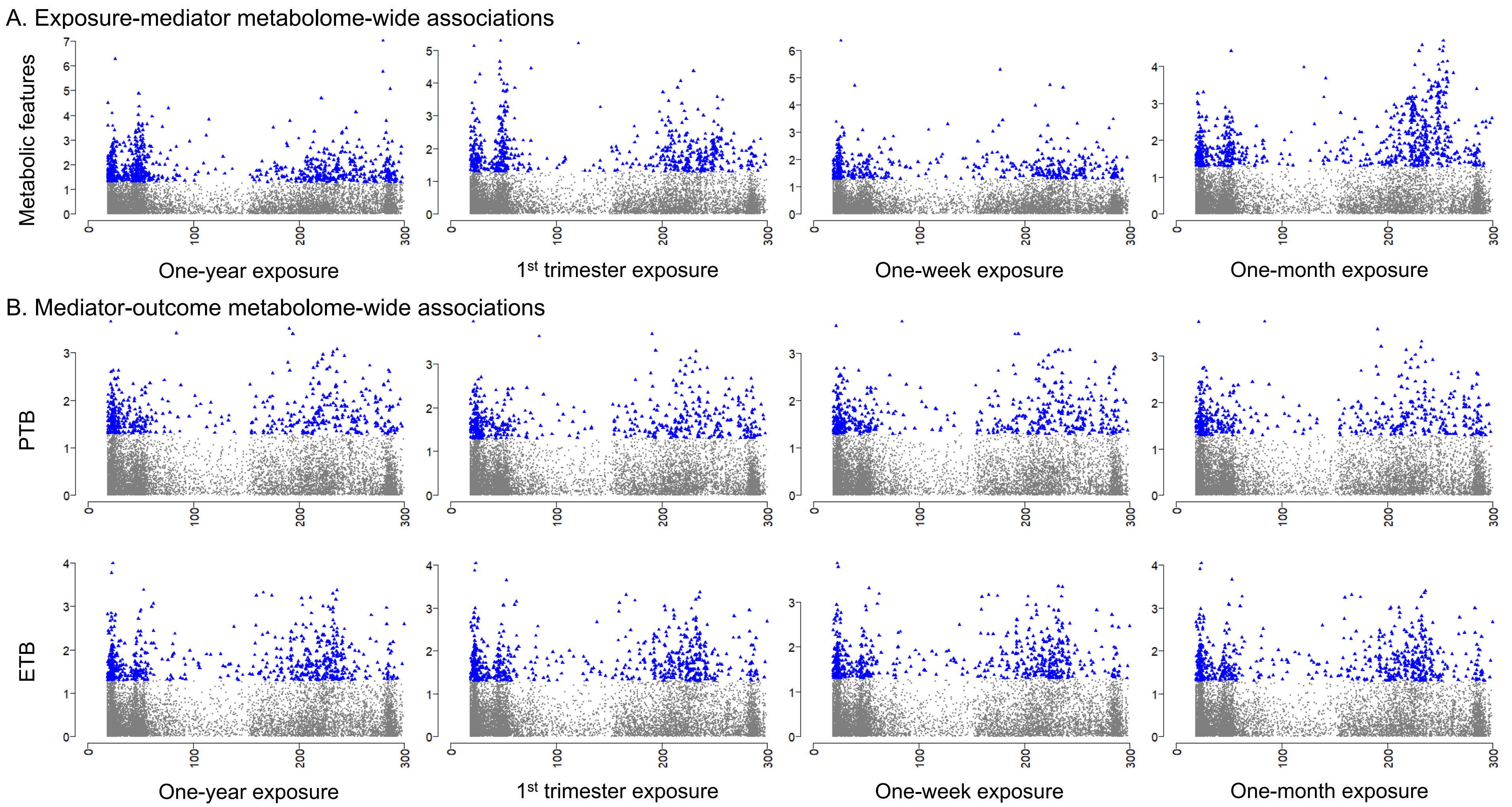

`

Figure S3. Manhattan plots of metabolome-wide association analysis in the C18 column. A. Associations between $PM_{2.5}$ exposures and changes in intensities of metabolic features; B. Associations between changes in intensities of metabolic features and PTB or ETB, with adjustment of $PM_{2.5}$ exposures for different time windows separately. X-axis denotes the retention time (in seconds) of the metabolic features, and Y-axis denotes the negative $\log_{10}$ of *p*-values. Red dots indicated significant associations at $FDR_{B-H} < 0.2$, and blue indicated associations at raw *p*-values < 0.05. Abbreviations: C18, hydrophobic reversed-phase chromatography; $PM_{2.5}$, fine particulate matter; PTB, preterm birth; ETB, early term birth; FDR_{B-H}, Benjamini-Hochberg adjusted *p*-values.

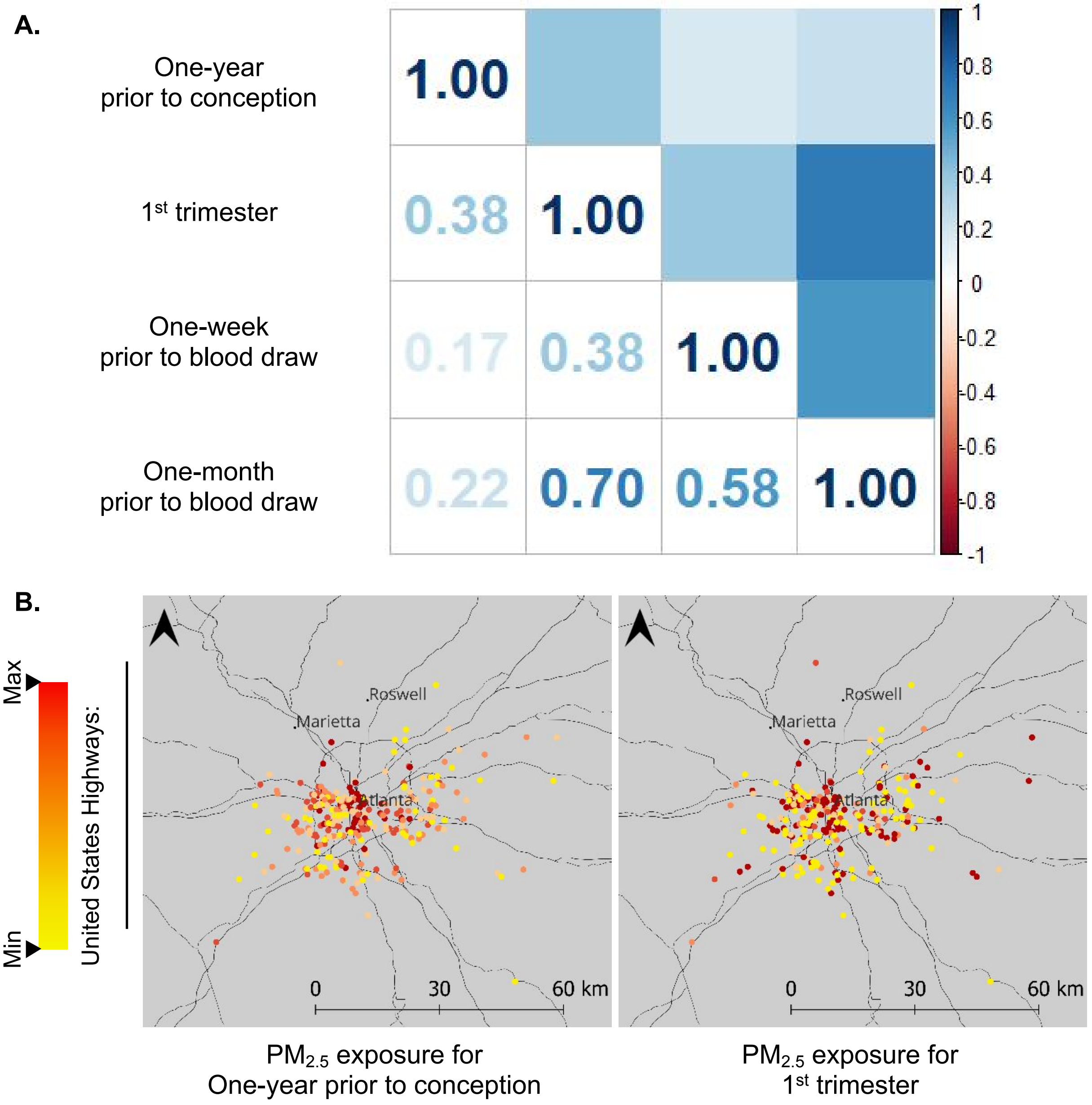

Figure S4. The Pearson's correlations among fine particulate matter ($PM_{2.5}$) exposures for the four exposure windows and the spatial variability of the $PM_{2.5}$ illustrated in Metropolitan Atlanta.

Table S1. Distribution of exposures to ambient fine particulate matter ($PM_{2.5}$, μg/m^3) in the four exposure windows and early birth outcomes by maternal marital status among 330 pregnant participants in the Atlanta African American Maternal-Child Cohort, 2014-2018.

	Married or cohabiting (N=157)	Not married or cohabiting (N=173)	*p*
One-year exposure prior to conception, mean (SD)	9.21 (0.72)	9.28 (0.97)	0.476
Exposure for the first trimester, mean (SD)	9.22 (1.31)	9.33 (1.54)	0.473
One-week exposure prior to blood draw, mean (SD)	9.06 (3.21)	9.28 (2.56)	0.482
One-month exposure prior to blood draw, mean (SD)	9.15 (1.96)	9.25 (1.88)	0.652
Early birth outcome, No. (%)			
Preterm	25 (15.9)	41 (23.7)	0.108
Early-term	31 (19.7)	23 (13.3)	
Full-term	101 (64.3)	109 (63.0)	

Table S2. Distribution of exposure to ambient fine particulate matter ($PM_{2.5}$) in the four exposure windows among 330 pregnant participants in the Atlanta African American Maternal-Child Cohort, 2014-2018.

$PM_{2.5}$ exposure (μg/m^3)	Q1	Q3	IQR	Median	Mean
One-year exposure prior to conception	8.76	9.69	0.93	9.27	9.24
Exposure for the first trimester	8.25	10.31	2.05	9.02	9.28
One-week exposure prior to blood draw	7.36	10.61	3.25	8.59	9.18
One-month exposure prior to blood draw	7.83	10.31	2.48	8.88	9.2

Abbreviations: Q1, 1st quartile; Q3, 3rd quartile; IQR, interquartile range.

Table S3. Number of metabolic features associated with $PM_{2.5}$ exposures or early birthoutcomes (preterm or early term birth) detected via the MWAS analysis among 330 pregnant participants in the Atlanta African American Maternal-Child Cohort, 2014-2018.

Exposures/Outcomes		Raw $p < 0.05$		$FDR_{B-H} < 0.2$	
		HILIC, ESI+	C18, ESI-	HILIC, ESI+	C18, ESI-
$PM_{2.5}$	1-year	918	854	97	67
	1st trimester	947	612	34	39
	1-week	553	465	2	6
	1-month	699	753	0	135
Preterm birth [a]	1-year	603	543	0	0
	1st trimester	618	548	0	0
	1-week	583	563	0	0
	1-month	596	543	0	0
Early term birth	1-year	1026	656	13	0
	1st trimester	1084	653	19	0
	1-week	1009	634	8	0
	1-month	1076	668	18	0

Abbreviations: $PM_{2.5}$, fine particulate matter; HILIC, hydrophilic interaction liquid chromatography; C18, hydrophobic reversed-phase chromatography; ESI, electrospray ionization.
[a] $PM_{2.5}$ of different exposure windows was also controlled individually in the mediator-outcome models in order to block the confounding resulting from the direct effect of $PM_{2.5}$, which resulted in four separately mediator-outcome models for each birth outcome.

Table S4. Model statistics of significant annotated metabolic features associated (adjusted $p < 0.2$) with ambient $PM_{2.5}$ exposures of different time windows among 330 pregnant participants in the Atlanta African American Maternal-Child Cohort, 2014-2018.

Column	Exposure	Metabolite	Coefficient [a]	Percent change (%) [b] (95% CI)	p	Adjusted p [c]
HILIC	1-year	Hydroxypyridine	0.138	14.8 (5.01, 25.5)	2.62×10^{-3}	0.191
		Quinoline	0.038	3.86 (1.32, 6.47)	2.96×10^{-3}	0.191
		Carnitine	0.051	5.24 (1.89, 8.71)	2.18×10^{-3}	0.191
	1st trimester	Phthalic anhydride	0.164	17.76 (9.08, 27.24)	3.73×10^{-5}	0.008
	1-week	Adenosine	-0.037	-3.66 (-5.74, -1.52)	9.64×10^{-4}	0.169
	1-month	Phthalic anhydride	0.105	11.04 (5.15, 17.25)	1.95×10^{-4}	0.044
		DEHP	0.071	7.41 (3.05, 11.95)	8.08×10^{-4}	0.090
		Lysope(20:3)	0.036	3.68 (1.26, 6.15)	2.92×10^{-3}	0.170
C18	1-year	Acetylcysteine	0.149	16.08 (5.75, 27.43)	1.88×10^{-3}	0.148
		ATP	0.035	3.54 (1.26, 5.85)	2.34×10^{-3}	0.148

Abbreviations: $PM_{2.5}$, fine particulate matter; HILIC, hydrophilic interaction liquid chromatography; C18, hydrophobic reversed-phase chromatography; CI, confidence interval; $FDR_{B\text{-}H}$, Benjamini-Hochberg adjusted *p*-values; ATP, adenosine triphosphate; DEHP, Bis(2-ethylhexyl) phthalate.

[a] Effect estimate associated with one-unit (μg/m^3) increase in $PM_{2.5}$.

[b] Feature intensity was natural-log transformed. Percent change was calculated by $(e^{\text{coefficient}}-1)\times100\%$.

[c] Multiple comparison correction was conducted via Benjamini-Hochberg procedure.

Table S5. Model statistics of significant annotated metabolic features associated (adjusted $p < 0.2$) with early term or preterm birth among 330 pregnant participant in the Atlanta African American Maternal-Child Cohort, 2014-2018.

Column	Metabolite	Outcome	Independent of exposure windows	Odds ratio (95% CI)	p	Adjusted p [a]
HILIC	Alanine	Early term	1-year	0.17 (0.05, 0.58)	4.42×10^{-3}	0.145
			1st trimester	0.14 (0.04, 0.49)	1.96×10^{-3}	0.119
			1-week	0.16 (0.05, 0.53)	2.74×10^{-3}	0.142
			1-month	0.16 (0.05, 0.53)	2.71×10^{-3}	0.123
	Choline	Early term	1-year	0.15 (0.04, 0.62)	8.32×10^{-3}	0.145
			1st trimester	0.16 (0.04, 0.62)	8.68×10^{-3}	0.148
			1-week	0.15 (0.04, 0.63)	9.20×10^{-3}	0.153
			1-month	0.16 (0.04, 0.64)	9.38×10^{-3}	0.149
	Proline	Early term	1-year	0.47 (0.28, 0.81)	6.22×10^{-3}	0.145
			1st trimester	0.46 (0.27, 0.79)	4.68×10^{-3}	0.119
			1-week	0.47 (0.28, 0.80)	5.69×10^{-3}	0.142
			1-month	0.47 (0.27, 0.79)	4.91×10^{-3}	0.123
	Taurine	Early term	1-month	0.29 (0.10, 0.82)	1.92×10^{-2}	0.199
	Hydroxyproline	Early term	1-year	0.44 (0.24, 0.80)	6.96×10^{-3}	0.145
			1st trimester	0.43 (0.24, 0.79)	6.48×10^{-3}	0.126
			1-week	0.47 (0.26, 0.85)	1.35×10^{-2}	0.197
			1-month	0.45 (0.25, 0.82)	8.87×10^{-3}	0.149
	Creatine	Early term	1-year	0.42 (0.22, 0.80)	7.98×10^{-3}	0.145
			1st trimester	0.40 (0.21, 0.76)	5.32×10^{-3}	0.119
			1-week	0.39 (0.20, 0.75)	4.95×10^{-3}	0.142
			1-month	0.41 (0.22, 0.79)	7.48×10^{-3}	0.149
	Leucine	Early term	1-year	0.14 (0.04, 0.53)	4.07×10^{-3}	0.145
			1st trimester	0.14 (0.04, 0.54)	4.14×10^{-3}	0.119
			1-week	0.14 (0.03, 0.53)	4.35×10^{-3}	0.142
			1-month	0.14 (0.04, 0.53)	3.74×10^{-3}	0.123
	Histidine	Early term	1-year	0.25 (0.09, 0.67)	5.89×10^{-3}	0.145
			1st trimester	0.25 (0.10, 0.67)	5.45×10^{-3}	0.119
			1-week	0.23 (0.08, 0.64)	4.77×10^{-3}	0.142
			1-month	0.24 (0.09, 0.65)	4.54×10^{-3}	0.123
	Carnitine	Early term	1st trimester	0.19 (0.05, 0.70)	1.29×10^{-2}	0.165
			1-month	0.19 (0.05, 0.72)	1.40×10^{-2}	0.175
	Phenylpyruvate	Early term	1-month	0.17 (0.04, 0.75)	1.93×10^{-2}	0.199
	Citrulline	Early term	1-year	0.37 (0.18, 0.79)	9.60×10^{-3}	0.145

			1st trimester	0.37 (0.17, 0.78)	9.31×10^{-3}	0.148
			1-week	0.35 (0.16, 0.75)	7.23×10^{-3}	0.153
			1-month	0.37 (0.17, 0.77)	8.47×10^{-3}	0.149
	Serotonin	Early term	1-year	0.40 (0.20, 0.82)	1.20×10^{-2}	0.162
			1st trimester	0.40 (0.20, 0.82)	1.20×10^{-2}	0.165
			1-week	0.38 (0.19, 0.79)	9.59×10^{-3}	0.153
			1-month	0.39 (0.19, 0.80)	1.05×10^{-2}	0.153
	Tyrosine	Early term	1-year	0.18 (0.05, 0.58)	4.23×10^{-3}	0.145
			1st trimester	0.17 (0.05, 0.56)	3.55×10^{-3}	0.119
			1-week	0.17 (0.05, 0.58)	4.20×10^{-3}	0.142
			1-month	0.17 (0.05, 0.55)	3.17×10^{-3}	0.123
	Cystine	Early term	1-year	0.39 (0.20, 0.77)	6.90×10^{-3}	0.145
			1st trimester	0.37 (0.19, 0.73)	4.05×10^{-3}	0.119
			1-week	0.41 (0.21, 0.81)	9.58×10^{-3}	0.153
			1-month	0.37 (0.19, 0.74)	4.59×10^{-3}	0.123
	Hexadecanol	Early term	1st trimester	2.17 (1.18, 4.01)	1.32×10^{-2}	0.165
	Cytidine 2',3'-cyclic monophosphate	Early term	1-year	0.59 (0.40, 0.88)	9.96×10^{-3}	0.145
			1st trimester	0.61 (0.41, 0.91)	1.52×10^{-2}	0.178
			1-month	0.61 (0.41, 0.90)	1.40×10^{-2}	0.175
	Cortexolone	Early term	1-year	0.58 (0.40, 0.83)	2.72×10^{-3}	0.145
			1st trimester	0.58 (0.41, 0.83)	3.16×10^{-3}	0.119
			1-week	0.56 (0.38, 0.80)	1.86×10^{-3}	0.142
			1-month	0.58 (0.40, 0.83)	3.11×10^{-3}	0.123
	Estriol	Early term	1-month	0.63 (0.43, 0.93)	1.83×10^{-2}	0.199
C18	Alpha-ketoisocaproate	Preterm	1-month	0.17 (0.05, 0.59)	4.72×10^{-3}	0.199
	Leucine	Preterm	1-month	0.16 (0.04, 0.57)	4.69×10^{-3}	0.199
	Methionine	Preterm	1-month	0.14 (0.04, 0.53)	3.60×10^{-3}	0.199
	Ascorbate	Early term	1-year	0.62 (0.46, 0.83)	1.51×10^{-3}	0.103
			1st trimester	0.63 (0.47, 0.85)	2.15×10^{-3}	0.136
			1-month	0.63 (0.47, 0.85)	2.10×10^{-3}	0.133
	Cytidine 2',3'-cyclic monophosphate	Early term	1-year	0.18 (0.06, 0.52)	1.62×10^{-3}	0.103
			1st trimester	0.19 (0.06, 0.53)	1.74×10^{-3}	0.136
			1-month	0.18 (0.06, 0.52)	1.53×10^{-3}	0.133

Abbreviations: $PM_{2.5}$, fine particulate matter; HILIC, hydrophilic interaction liquid chromatography; C18, hydrophobic reversed-phase chromatography; CI, confidence interval; $FDR_{B\text{-}H}$, Benjamini-Hochberg adjusted *p*-values.

[a] Multiple comparison correction was conducted via Benjamini-Hochberg procedure.

Table S6. Biological pathways associated with $PM_{2.5}$ exposures detected by the pathway enrichment analysis.

Biological pathways	Exposure windows [a]	Number of significant metabolites	Number of total metabolites	*P*-values
Estrone metabolism	1-year	2	24	0.037
Phenylalanine, tyrosine and tryptophan biosynthesis	1-year	2	19	0.029
Purine metabolism	1-year	10	108	0.006
Serotonergic synapse	1-year	2	38	0.043
Arachidonic acid metabolism	1-year	4	103	0.015
TCA cycle	1-year	3	26	0.006
Tryptophan metabolism	1-year	6	94	0.045
Retinol metabolism	1st trimester	3	42	0.018
Phenylalanine metabolism	1-week	2	47	0.003
Protein digestion and absorption	1-week	4	43	0.016
Tryptophan metabolism	1-week	7	94	0.001
Biopterin metabolism	1-week	2	16	0.008

[a] Exposure windows include one-year prior to conception, the first trimester, one-week and one-month prior to blood draw.

Table S7. Biological pathways associated with early birth outcomes [i.e., preterm birth (PTB) and early-term birth (ETB)] detected by the pathway enrichment analysis, with adjusting for $PM_{2.5}$ exposures.

Biological pathways	Outcome	Controlled mediator	Number of significant metabolites	Number of total metabolites	*P*-values
2-oxocarboxylic acid metabolism	PTB	1-year	4	49	0.018
Aminoacyl-trna biosynthesis	PTB	1-year	2	23	0.031
Parkinson's disease	PTB	1-year	2	14	0.004
Protein digestion and absorption	PTB	1-year	4	43	0.006
2-oxocarboxylic acid metabolism	PTB	1st trimester	4	49	0.026
Aminoacyl-trna biosynthesis	PTB	1st trimester	2	23	0.043
Parkinson's disease	PTB	1st trimester	2	14	0.010
Protein digestion and absorption	PTB	1st trimester	4	43	0.013
Carnitine shuttle	PTB	1st trimester	2	45	0.049
Protein digestion and absorption	PTB	1-week	4	43	0.012
2-oxocarboxylic acid metabolism	PTB	1-month	4	49	0.008
Aminoacyl-trna biosynthesis	PTB	1-month	2	23	0.030
Parkinson's disease	PTB	1-month	2	14	0.005
Protein digestion and absorption	PTB	1-month	4	43	0.005
Carnitine shuttle	PTB	1-month	2	45	0.046
2-oxocarboxylic acid metabolism	ETB	1-year	4	49	0.029
Cysteine and methionine metabolism	ETB	1-year	6	45	0.031
Fatty acid degradation	ETB	1-year	2	40	0.006
Galactose metabolism	ETB	1-year	4	56	0.036
Glycerophospholipid metabolism	ETB	1-year	9	70	0.000
Glycine and serine metabolism	ETB	1-year	8	56	0.001
Glycine, serine and threonine metabolism	ETB	1-year	5	41	0.009
Glycine, serine, alanine and threonine metabolism	ETB	1-year	10	86	0.002
Methane metabolism	ETB	1-year	4	47	0.037
Mineral absorption	ETB	1-year	3	26	0.005
Aminoacyl-trna biosynthesis	ETB	1-year	4	23	0.007
Neuroactive ligand-receptor interaction	ETB	1-year	4	48	0.046
Pantothenate and coa biosynthesis	ETB	1-year	3	31	0.012
Pentose and glucuronate interconversions	ETB	1-year	2	33	0.039
Phenylalanine, tyrosine and tryptophan biosynthesis	ETB	1-year	2	19	0.045
Porphyrin and chlorophyll metabolism	ETB	1-year	3	40	0.035
Protein digestion and absorption	ETB	1-year	7	43	0.000
Abc transporters	ETB	1-year	9	73	0.000
Selenocompound metabolism	ETB	1-year	3	15	0.014
Sialic acid metabolism	ETB	1-year	3	35	0.003
Taste transduction	ETB	1-year	3	28	0.001
Taurine and hypotaurine metabolism	ETB	1-year	3	23	0.014
Valine, leucine and isoleucine biosynthesis	ETB	1-year	2	18	0.000
Aspartate and asparagine metabolism	ETB	1-year	7	82	0.019
Beta-alanine metabolism	ETB	1-year	4	51	0.034
Biosynthesis of amino acids	ETB	1-year	8	92	0.046
Central carbon metabolism in cancer	ETB	1-year	4	36	0.009
2-oxocarboxylic acid metabolism	ETB	1st trimester	4	49	0.018
Cysteine and methionine metabolism	ETB	1st trimester	6	45	0.041

Biological pathways	Outcome	Controlled mediator	Number of significant metabolites	Number of total metabolites	*P*-values
Fatty acid degradation	ETB	1st trimester	2	40	0.009
Galactose metabolism	ETB	1st trimester	4	56	0.019
Glycerolipid metabolism	ETB	1st trimester	3	31	0.036
Glycerophospholipid metabolism	ETB	1st trimester	9	70	0.002
Glycine and serine metabolism	ETB	1st trimester	8	56	0.002
Glycine, serine and threonine metabolism	ETB	1st trimester	6	41	0.007
Glycine, serine, alanine and threonine metabolism	ETB	1st trimester	10	86	0.002
Mineral absorption	ETB	1st trimester	4	26	0.001
Aminoacyl-trna biosynthesis	ETB	1st trimester	4	23	0.004
Neuroactive ligand-receptor interaction	ETB	1st trimester	4	48	0.045
Pantothenate and coa biosynthesis	ETB	1st trimester	4	31	0.008
Propanoate metabolism	ETB	1st trimester	4	64	0.046
Protein digestion and absorption	ETB	1st trimester	7	43	0.000
ABC transporters	ETB	1st trimester	10	73	0.000
Selenoamino acid metabolism	ETB	1st trimester	4	34	0.013
Selenocompound metabolism	ETB	1st trimester	4	15	0.000
Sialic acid metabolism	ETB	1st trimester	3	35	0.001
Taste transduction	ETB	1st trimester	3	28	0.000
Taurine and hypotaurine metabolism	ETB	1st trimester	3	23	0.021
Valine, leucine and isoleucine biosynthesis	ETB	1st trimester	3	18	0.000
Biosynthesis of amino acids	ETB	1st trimester	8	92	0.030
Central carbon metabolism in cancer	ETB	1st trimester	4	36	0.006
Cysteine and methionine metabolism	ETB	1-week	6	45	0.028
Fatty acid degradation	ETB	1-week	2	40	0.006
Galactose metabolism	ETB	1-week	4	56	0.036
Glycerophospholipid metabolism	ETB	1-week	8	70	0.008
Glycine and serine metabolism	ETB	1-week	7	56	0.003
Glycine, serine and threonine metabolism	ETB	1-week	5	41	0.012
Glycine, serine, alanine and threonine metabolism	ETB	1-week	9	86	0.014
Mineral absorption	ETB	1-week	3	26	0.011
Aminoacyl-trna biosynthesis	ETB	1-week	3	23	0.010
Pantothenate and coa biosynthesis	ETB	1-week	3	31	0.024
Porphyrin and chlorophyll metabolism	ETB	1-week	4	40	0.005
Porphyrin metabolism	ETB	1-week	3	47	0.049
Protein digestion and absorption	ETB	1-week	7	43	0.000
ABC transporters	ETB	1-week	9	73	0.000
Selenocompound metabolism	ETB	1-week	3	15	0.009
Sialic acid metabolism	ETB	1-week	3	35	0.004
Squalene and cholesterol biosynthesis	ETB	1-week	2	44	0.040
Taste transduction	ETB	1-week	3	28	0.001
Taurine and hypotaurine metabolism	ETB	1-week	3	23	0.010
Valine, leucine and isoleucine biosynthesis	ETB	1-week	2	18	0.002
Aspartate and asparagine metabolism	ETB	1-week	6	82	0.040
Biosynthesis of amino acids	ETB	1-week	8	92	0.042
Central carbon metabolism in cancer	ETB	1-week	4	36	0.023
2-oxocarboxylic acid metabolism	ETB	1-month	4	49	0.025

Biological pathways	Outcome	Controlled mediator	Number of significant metabolites	Number of total metabolites	*P*-values
Cysteine and methionine metabolism	ETB	1-month	6	45	0.037
Fatty acid degradation	ETB	1-month	2	40	0.004
Galactose metabolism	ETB	1-month	4	56	0.031
Glycerophospholipid metabolism	ETB	1-month	9	70	0.001
Glycine and serine metabolism	ETB	1-month	8	56	0.002
Glycine, serine and threonine metabolism	ETB	1-month	5	41	0.011
Glycine, serine, alanine and threonine metabolism	ETB	1-month	10	86	0.004
Methane metabolism	ETB	1-month	4	47	0.038
Mineral absorption	ETB	1-month	3	26	0.005
Aminoacyl-trna biosynthesis	ETB	1-month	4	23	0.004
Neuroactive ligand-receptor interaction	ETB	1-month	4	48	0.018
Pantothenate and coa biosynthesis	ETB	1-month	3	31	0.012
Phenylalanine, tyrosine and tryptophan biosynthesis	ETB	1-month	2	19	0.032
Porphyrin and chlorophyll metabolism	ETB	1-month	3	40	0.042
Protein digestion and absorption	ETB	1-month	7	43	0.000
ABC transporters	ETB	1-month	9	73	0.001
Selenocompound metabolism	ETB	1-month	3	15	0.010
Sialic acid metabolism	ETB	1-month	3	35	0.002
Taste transduction	ETB	1-month	3	28	0.000
Taurine and hypotaurine metabolism	ETB	1-month	3	23	0.012
Valine, leucine and isoleucine biosynthesis	ETB	1-month	2	18	0.001
Biosynthesis of amino acids	ETB	1-month	8	92	0.028
Central carbon metabolism in cancer	ETB	1-month	4	36	0.002

[a] Exposure windows include one-year prior to conception, the first trimester, one-week and one-month prior to blood draw. $PM_{2.5}$ exposure was adjusted to block the direct path from $PM_{2.5}$ to the outcome.

Table S8. Number of metabolic features associated with $PM_{2.5}$ exposures ($FDR_{B-H} < 0.2$) with or without adjusting for hypertension disorders of pregnancy and gestational diabetes, detected via the MWAS analysis among 330 pregnant participants in the Atlanta African American Maternal-Child Cohort, 2014-2018.

Exposures		Model without pregnancy complications		Model with hypertension disorders		Model with Gestational diabetes	
		HILIC, ESI+	C18, ESI-	HILIC, ESI+	C18, ESI-	HILIC, ESI+	C18, ESI-
$PM_{2.5}$	1-year	97	67	102	57	99	66
	1st trimester	34	39	30	30	29	39
	1-week	2	6	3	6	2	6
	1-month	0	135	0	139	0	125

FDR_{B-H}, false discovery rate of Benjamini-Hochberg procedure.

Table S9. Number of metabolic features associated with $PM_{2.5}$ exposures ($FDR_{B-H} < 0.2$) with adjusting for apparent temperature or natural smooth function of relative humidity and temperature, detected via the MWAS analysis among 330 pregnant participants in the Atlanta African American Maternal-Child Cohort, 2014-2018.

Exposures		Model with apparent temperature		Model with natural smooth function [a] of relative humidity and temperature	
		HILIC, ESI+	C18, ESI-	HILIC, ESI+	C18, ESI-
$PM_{2.5}$	1st trimester	34	39	40	77
	1-week	2	6	6	6
	1-month	0	135	16	125

FDR_{B-H}, false discovery rate of Benjamini-Hochberg procedure.

[a] Natural spline terms with 3 degrees of freedom.

Table S10. Number of overlapping metabolic features associated $PM_{2.5}$ exposures and early birth, detected via the MWAS analysis among 330 pregnant participants in the Atlanta African American Maternal-Child Cohort, 2014-2018.

Outcome	Exposure	Raw $p < 0.05$		$FDR_{B-H} < 0.2$	
		HILIC, ESI+	C18, ESI-	HILIC, ESI+	C18, ESI-
Preterm birth	1-year	34	45	0	0
	1st trimester	50	47	0	0
	1-week	50	37	0	0
	1-month	46	58	0	0
Early term birth	1-year	113	74	0	0
	1st trimester	73	58	0	0
	1-week	63	37	0	0
	1-month	64	85	0	0

Abbreviations: $PM_{2.5}$, fine particulate matter; HILIC, hydrophilic interaction liquid chromatography; C18, hydrophobic reversed-phase chromatography; ESI, electrospray ionization.

Table S11. The significant metabolites mapped in the overlapping biological pathways associated with PM2.5 exposures and early birth outcomes detected by the pathway enrichment analysis.

Pathway	Exposure/outcome associated	HMDB identification of mapped metabolites
Protein digestion and absorption	1-week $PM_{2.5}$ exposure	HMDB0000182, HMDB0000929, HMDB0000158, HMDB0000687, HMDB0000162, HMDB0000883, HMDB0000172, HMDB0000738, HMDB0034301
	PTB	HMDB0000051, HMDB0000696, HMDB0000929, HMDB0000159, HMDB0000158, HMDB0000687, HMDB0000883, HMDB0000172, HMDB0034301, HMDB0000718, HMDB0002176
	ETB	HMDB0000161, HMDB0000182, HMDB0000517, HMDB0000187, HMDB0000929, HMDB0000158, HMDB0000056, HMDB0000687, HMDB0000177, HMDB0000162, HMDB0000237, HMDB0000883, HMDB0000172, HMDB0000192, HMDB0000588, HMDB0034301, HMDB0000718, HMDB000217
Phenylalanine, tyrosine, and tryptophan biosynthesis	1-year $PM_{2.5}$ exposure	HMDB0000263, HMDB0000929, HMDB0000205, HMDB0000738, HMDB0003070, HMDB0002466, HMDB0012710, HMDB0000707
	ETB	HMDB0000263, HMDB0000929, HMDB0000158, HMDB0000205, HMDB0012249, HMDB0000738, HMDB0001076

Table S12. Model statistics of the significant mediating metabolites (adjusted p-value < 0.2 via Benjamini-Hochberg procedure) between $PM_{2.5}$ exposure and early birth detected by high-dimensional mediation analysis.

Metabolite	Exposure	Outcome	α [a]	β [b]	$\alpha \times \beta$ [c]	Proportion mediated [d]	Adjusted p value
Alpha-ketoisocaproate	1-week	Preterm	0.008	-0.634	-0.005	51.8%	0.121
	1-month	Preterm	0.011	-0.450	-0.005	-4.5%	0.184
Leucine	1-month	Preterm	0.018	-0.531	-0.009	-8.2%	0.140
	1-week	Early term	0.011	-1.305	-0.015	9.1%	0.043
Proline	1-year	Preterm	0.073	-1.58×10^{-4}	-1.16×10^{-5}	-0.01%	0.077
Pyridoxamine	1-year	Early term	0.066	-0.294	-0.019	6.8%	0.140
	1st trimester	Early term	0.039	-0.384	-0.015	10.5%	0.039
	1-week	Early term	0.024	-0.192	-0.005	2.8%	0.063
Tyrosine	1-month	Preterm	0.015	-0.850	-0.013	-11.5%	0.105
N-acetyl-d-galactosamine	1st trimester	Preterm	-0.042	0.430	-0.018	-9.0%	0.035
	1-month	Preterm	-0.030	0.822	-0.025	-21.6%	0.079
Cortexolone	1-week	Preterm	-0.039	-0.401	0.016	155.1%	0.019
	1-week	Early term	-0.041	-0.459	0.019	-11.8%	0.043
Lysope(20:3)	1-month	Preterm	0.026	0.407	0.011	9.4%	0.079

Abbreviations: $PM_{2.5}$, fine particulate matters.

[a] Coefficient estimates of the association between exposure and mediators calculated by the R package *HIMA*. Alpha represents the effect estimate of $PM_{2.5}$ regrading one μg/m^3 increase on the metabolite intensity.

[b] Coefficient estimates of the association between mediators and outcome (adjusted for exposure) calculated by the R package *HIMA*, which was derived from the multiple-mediator-outcome model. Beta represents the effect estimate of the metabolite on the risk of PTB/ETB, independent of other metabolites.

[c] Estimated mediation effect.

[d] Negative proportion mediated occurs when the direct and indirect effects have opposite signs. The proportion mediated may exceed 100% if there are other mediators with a "negative" proportion mediated.
